# Mesoporous PU/PEDOT:PSS Electrodes Reveal Population‐Level Stochastic Bioelectrical Dynamics in Marine Diatoms

**DOI:** 10.1002/advs.77341

**Published:** 2026-09-05

**Authors:** David M. S. Silva, Felipe L. Bacellar, Raquel Amaral, Louis‐Josselin Lavier‐Aydat, Luísa Cortes, Kamal Asadi, Leïla Tirichine, Paulo R. F. Rocha

**Affiliations:** ^1^ Bioelectronics & Bioenergy Research Lab Centre For Functional Ecology‐Science for People & the Planet, Associate Laboratory TERRA Department of Life Sciences University of Coimbra Coimbra Portugal; ^2^ CNRS US2B Nantes Université Nantes France; ^3^ CNC‐UC – Center for Neuroscience and Cell Biology and CiBB – Center for Innovative Biomedicine and Biotechnology University of Coimbra Coimbra Portugal; ^4^ Department of Physics University of Bath Bath, Claverton Down UK; ^5^ Institute for Marine and Antarctic Studies (IMAS) Ecology and Biodiversity Centre University of Tasmania Hobart TAS Australia

**Keywords:** diatom ecophysiology, mesoporous electrodes, microalgae, mixed ionic‐electronic conduction, PEDOT:PSS, stochastic bioelectrochemistry

## Abstract

Understanding how microbial populations generate and modulate electrical signals remains a major technical challenge because extracellular ionic processes are inherently weak, spatially distributed and stochastic. Here, we introduce a mesoporous ultra‐low‐impedance PEDOT:PSS electrode that exploits volumetric ionic‐electronic coupling to probe stochastic electrochemical dynamics in axenic populations of the marine model diatom *Phaeodactylum tricornutum*. The three‐dimensional porous architecture provides a large electrochemically accessible surface area with high capacitance and low impedance, enabling sensitive detection of non‐equilibrium current fluctuations under applied bias. Applied bias systematically amplifies stochastic electrical fluctuations, while living diatoms significantly modify their amplitude and temporal statistics. Noise analysis reveals enhanced low‐frequency fluctuations, increased power‐law exponents and higher transient‐event rates, indicating biologically driven perturbations of the local electrochemical environment. Pharmacological inhibition with tetraethylammonium suppresses spontaneous electrical activity, providing independent functional evidence that membrane‐associated potassium‐dependent processes contribute to the measured signals. Increasing diatom cell density further produces progressively larger spectral exponents and distinct changes in stochastic‐event dynamics, demonstrating that these electrical signatures encode microbial population density. These findings establish stochastic electrochemical noise as a sensitive, label‐free probe of microbial bioelectrochemical activity and a bioelectronic strategy for monitoring living microbial systems through their intrinsic non‐equilibrium electrical fluctuations.

## Graphical abstract

Mesoporous mixed ionic‐electronic PU/PEDOT: PSS electrodes enable label‐free detection of stochastic extracellular bioelectrical dynamics in living *Phaeodactylum tricornutum*. Living diatoms actively modulate interfacial ionic dynamics, establishing stochastic electrochemical noise as a sensitive probe of microbial bioelectrochemical activity and a new bioelectronic strategy for monitoring living microbial systems.

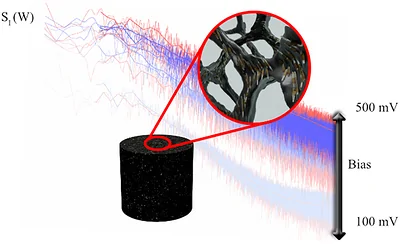

## 1 Introduction

Bioelectrical phenomena are fundamental to living systems, from single ion channels in unicellular species to complex multicellular networks. While electrophysiology has historically focused on excitable systems such as neurons and cardiac tissue, an increasing body of evidence indicates that non‐excitable microorganisms also exhibit electrochemical dynamics, arising from ionic transport, membrane fluctuations and concomitant metabolic activity. However, accessing these electroactive signalling mechanisms in environmentally relevant biological systems remains a major challenge, largely due to limitations in how bioelectronic interfaces couple to ionic media. Additionally, conventional electrophysiological approaches are inherently surface‐confined, probing localized electrical activity through planar or microfabricated electrodes. While highly successful in neuroscience and cellular electrophysiology, these strategies are not optimized to interrogate spatially distributed, weak and stochastic signals emerging from complex biological media, particularly due to the large electrochemical impedance inherent from small microfabricated electrodes [1]. Thus, an entire class of bioelectrical dynamics, particularly those arising from collective ionic fluctuations and distributed interfacial processes in microbial populations remains largely unexplored.

In parallel, studies across physics, biology and neuroscience have demonstrated that biological systems frequently exhibit scale‐invariant fluctuations, often described by power spectral densities (PSDs) following a 1/f dependence, suggesting that noise is not merely a measurement artifact, but rather a fundamental signal reflecting the underlying organization and dynamics of living systems [1, 2, 3]. While noise has been linked to intrinsic ion channel activity, distributed noise sources and multiscale system dynamics, noise analysis remains largely absent from bioelectronic measurements of non‐excitable microorganisms, where it could provide unique access to otherwise hidden electrochemical processes. Per instance, in non‐equilibrium electronic and electrochemical systems, low‐frequency spectra have shown to deviate from the canonical 1/f form and approach exponents near 3/2, a behavior routinely associated with diffusion‐like fluctuations and Brownian transport processes [3, 4].

Photosynthetic microorganisms represent a particularly compelling yet underexplored class of biological systems. Among them, the marine diatom *Phaeodactylum tricornutum* has become an established model organism [5] due to its genetic tractability, well‐characterized physiology and ecological relevance as a member of the diatoms, one of the most important group of marine primary producers [6]. Existing techniques for studying diatoms, including optical spectroscopy, fluorescence imaging and electrochemical assays, provide valuable insights into metabolic activity, but they do not capture the dynamic electrical signatures associated with ionic transport and membrane processes. Increasing evidence suggests that diatoms possess ion‐transport mechanisms and membrane‐associated processes capable of supporting electrical activity, although these have been investigated primarily through biochemical [7, 8], genetic [9] or optical approaches [10, 11, 12]. In addition, intracellular electrophysiological studies have demonstrated that certain diatom species can generate fast Na^+^/Ca^2+^ action potentials‐like signals under controlled stimulation conditions, revealing the presence of voltage‐gated ion channels and membrane excitability at the single‐cell level [13]. However, these approaches rely on invasive intracellular recording configurations and probe isolated cellular responses rather than the collective electrical dynamics of cell populations in their native aqueous environment. Complementary studies using planar electrode configurations have reported electrical oscillations in small diatom populations, typically under stress conditions, suggesting coordinated ionic responses [14]. Nevertheless, these measurements remain limited in sensitivity and primarily capture synchronized or quasi‐periodic activity under stress. As a result, real‐time, high‐sensitivity electrical interrogation of stochastic, unsynchronized ionic dynamics in diatom populations under fully submerged conditions remains largely unexplored. Existing techniques do not accurately capture how diatom‐electrode systems modulate distributed charge transport, interfacial impedance and noise characteristics within three‐dimensional conducting networks. This represents a critical gap in understanding how ionic fluxes couple to electronic transport in biohybrid systems [15].

Although porous PU/PEDOT:PSS electrodes have previously been employed to investigate collective electrical activity in filamentous cyanobacteria [2], extending this approach to diatoms presents distinct scientific and technological challenges. In contrast to filamentous cyanobacteria, *Phaeodactylum tricornutum* is a unicellular eukaryotic microalga that retains characteristic diatom cell‐envelope features, including a silica‐based frustule whose degree of silicification depends on growth conditions, together [16] with extracellular polymeric substances (EPS) [17]. These surface structures substantially influence cell‐electrode interactions, ionic transport [14] and interfacial electrochemistry. Furthermore, the origin and statistical properties of electrical fluctuations in diatom populations remain essentially unexplored. These differences motivated the development of an improved porous PEDOT:PSS platform with enhanced electrical reproducibility and stability, enabling quantitative stochastic analysis of bioelectrochemical activity over extended measurement periods. Hence, we introduce an ultra‐low‐impedance electrochemical interface based on mesoporous poly(3,4‐ethylenedioxythiophene):poly(styrene sulfonate) PEDOT:PSS electrodes that enables distributed ionic‐electronic coupling in aqueous environments. We hypothesize that such volumetric mixed conductors give rise to non‐equilibrium electrochemical dynamics that manifest as stochastic electrical fluctuations under applied bias. Using axenic cultures of the model marine diatom *Phaeodactylum tricornutum* accession 15 (Pt15), we demonstrate that these fluctuations that exceed thermal‐noise, exhibit power‐law spectral behavior and are systematically modulated by biological activity.

## 2 Results and Discussion

### 2.1 Porous PEDOT:PSS Electrode Architecture and Electrical Conductivity

The porous electrodes used in this study were fabricated by infiltrating laser‐cut polyurethane (PU) scaffolds with PEDOT:PSS through a vacuum‐assisted dip‐coating process (Figure 1A). The open‐cell structure of the PU scaffold provides a three‐dimensional template in which the conducting polymer forms a continuous mixed ionic‐electronic conducting network throughout the porous volume. This architecture significantly increases the effective electrochemical interface compared with planar thin‐film electrodes. Electrical transport measurements were first performed to evaluate the formation of conductive pathways within the scaffold. Current‐voltage (I‐V) measurements between copper contacts attached to opposite faces of the electrode (see Figure 1A‐VII and Figure S1) reveal that the conductivity of the scaffold increases with the number of dip‐coating cycles, over one order of magnitude compared to previous published PU/PEDOT:PSS foams [2]. A single coating cycle produces only partial electronic connectivity through the scaffold, resulting in relatively high resistance. Although the measured current‐voltage characteristics remain approximately linear, indicating ohmic transport, the limited connectivity suggests that the conducting polymer network is not yet fully percolated throughout the porous structure. Increasing the number of coating cycles leads to progressive infiltration of PEDOT:PSS throughout the pore network, enabling the formation of continuous conductive pathways.

**FIGURE 1:**
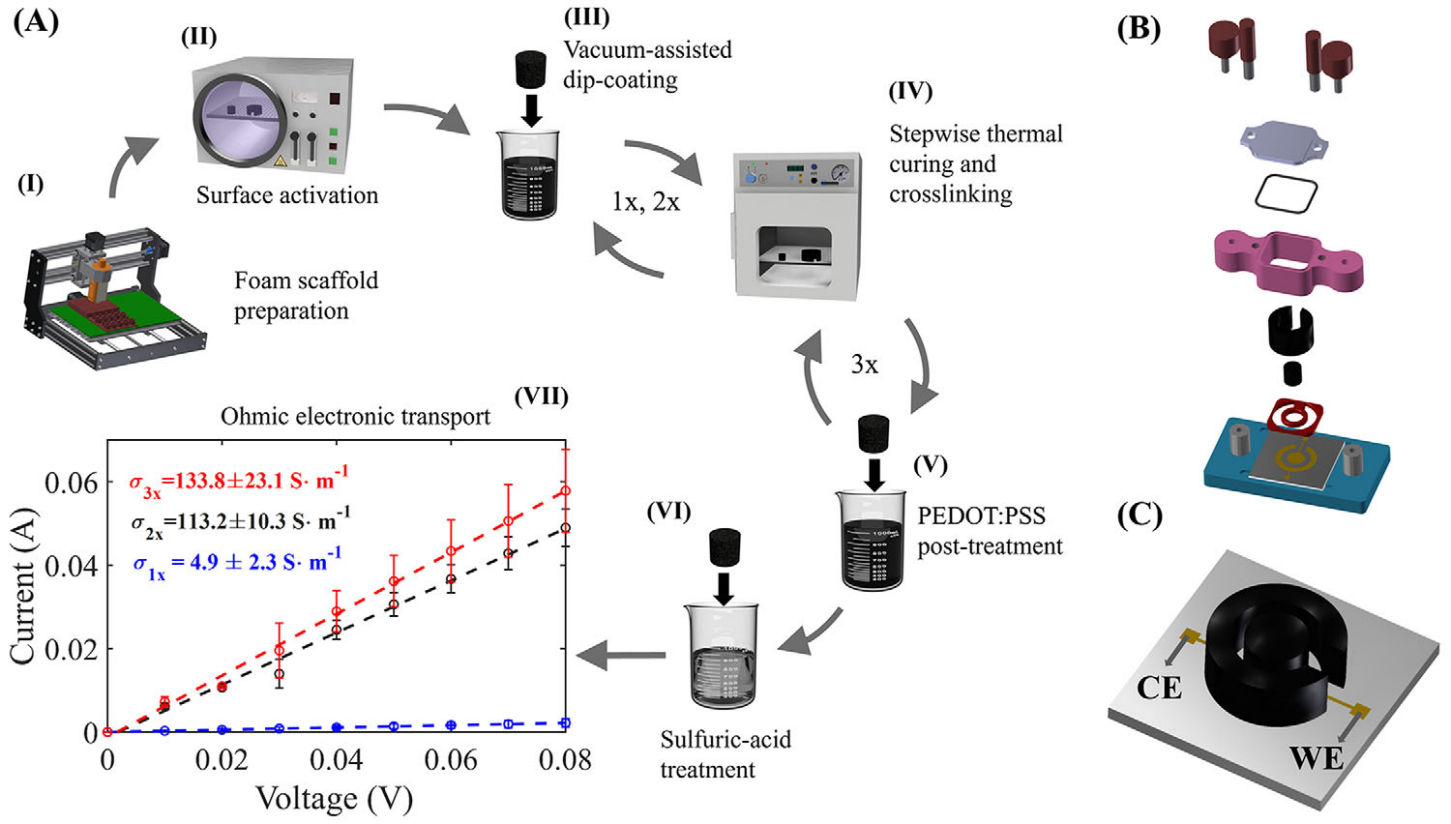
Fabrication, assembly and electronic characterization of mesoporous PU/PEDOT:PSS electrodes. (A) Schematic of the fabrication workflow for porous mixed ionic‐electronic conductor electrodes. (I) Polyurethane (PU) foam scaffolds are laser‐cut into cylindrical geometries. (II) Surface activation by oxygen plasma treatment enhances wettability and coating adhesion. (III) PEDOT:PSS infiltration is achieved by vacuum‐assisted dip‐coating using an ethylene‐glycol‐ and PEG‐crosslinker‐modified formulation. (IV) Stepwise thermal curing induces solvent removal, film consolidation and crosslinking within the porous network. The dip‐coating and curing cycle is repeated to obtain three coating cycles (3x). (V) A solvent post‐treatment using polar co‐solvents promotes polymer chain reorganization and electronic percolation. (VI) A sulfuric‐acid post‐treatment removes excess insulating PSS and enhances electronic conductivity, yielding the optimized 3x acid‐treated PU/PEDOT:PSS electrodes used throughout this work. (VII) Representative current‐voltage characteristics demonstrate linear ohmic electronic transport and a systematic increase in effective conductivity with coating number. (B) Exploded schematic of the sealed electrode holder and current collector assembly designed to maintain a closed, mechanically stable and contamination‐free environment during measurements. (C) Assembled working electrode (WE) and counter electrode (CE) configuration used for electrochemical measurements.

After three dip‐coating cycles, the electrode conductivity increases by approximately two orders of magnitude compared with the single‐coated scaffold. This improvement indicates that the PEDOT:PSS forms a well‐connected electronic network throughout the porous structure. Additional coating cycles beyond three do not significantly improve conductivity and can instead increase resistive contributions due to excessive polymer accumulation and partial blockage of the pore network (see Figure S3). Independent structural characterization using mercury porosimetry further shows that the optimized electrodes retain high porosity and an open pore network after coating and acid post‐treatment (Figures S2 and S4), supporting efficient electrolyte penetration. As shown in Figure S2, the median pore diameter decreases relative to the uncoated scaffold, consistent with conformal coating of the pore walls by PEDOT:PSS, whereas the overall porosity remains high, at around 60%. Importantly, the final sulfuric‐acid post‐treatment does not measurably alter pore diameter or porosity relative to the three‐coating state, indicating that the pore‐network integrity is preserved during conductivity enhancement. This configuration is particularly advantageous for bioelectronic measurements because it enables efficient coupling between electronic charge transport in the electrode and ionic transport in the surrounding electrolyte. Figure 1B shows the full solution holder apparatus comprising a top‐lid to provide a contamination‐free environment during measurements. Figure 1C shows a 3D image of the working electrode (WE) and counter electrode (CE) PU/PEDOT:PSS mesoporous foams assembled on a glass substrate with thermally evaporated Au collectors. After establishing a mesoporous volumetric mixed conductor, we examined its electrochemical performance.

### 2.2 Ultra‐low Electrochemical Impedance Response of Mesoporous PEDOT:PSS Electrodes

Impedance response reveals low‐impedance, high‐capacitance and distributed behavior. The electrochemical properties of the porous PU/PEDOT:PSS electrodes were characterized using electrochemical impedance spectroscopy (EIS) in artificial seawater (EASW). The impedance spectra were fitted using an equivalent circuit consisting of a solution resistance in series with a parallel combination of a charge‐transfer resistance and a constant phase element (Rs + (Rct || CPE)), representing the electrolyte resistance, interfacial charge‐transfer processes and non‐ideal capacitive behavior of the porous PU/PEDOT:PSS interface. The impedance response can therefore be expressed as:

(1) $$Z\left(\omega \right)=R_{s}+\frac{1}{\frac{1}{R_{ct}}+{\left(j\omega \right)}^{n}Q}$$

where *R_s_* represents the solution resistance, *R_ct_* the charge transfer resistance, and *Q* and *n* describe the constant phase element (CPE) representing the interfacial capacitance.

Figure 2 shows the impedance magnitude and phase for electrodes fabricated with different numbers of dip‐coating cycles. Increasing the number of coating cycles reduces the impedance magnitude across the entire frequency range and improves the interface capacitance. The optimized three‐cycle electrodes display the lowest impedance and the most stable capacitive response. Equivalent circuit fitting reveals that increasing the coating number reduces both the series resistance *R_s_* and the charge transfer resistance *R_ct_*, while simultaneously increasing the effective interfacial capacitance derived from the CPE parameters (Tables 1 and 2). Notably, the extracted areal capacitance (Table 2) falls within the range reported for state‐of‐the‐art PEDOT:PSS electrodes [18, 19] (∼1 mF cm^−2^), supporting that the present system operates within the expected electrochemical regime of volumetric charge storage mediated by ion‐accessible surface area. This agreement validates both the impedance analysis and the effective electrochemical accessibility of the porous network. The increase in capacitance reflects the large electrochemically accessible surface area of the porous network. Importantly, the exponent *n* of the constant phase element remains close to unity (*n* ≈ 0.92‐0.95), indicating that the interface behaves close to an ideal capacitor. This behavior confirms that the porous PEDOT:PSS network forms a stable mixed ionic‐electronic interface that supports efficient ion exchange with the electrolyte and reflects distributed electrochemical processes, where different regions of the porous network contribute with distinct time constants. Also, because the impedance spectrum directly determines the dissipative admittance of the interface as:

(2) $$Y\left(\omega \right)=\frac{1}{Z\left(\omega \right)}\;$$

it also defines the equilibrium thermal current noise expected from the Johnson–Nyquist relation:

(3) SIf=4kTReYf

where *k* is Boltzmann's constant, and *T* is the absolute temperature. This thermal noise prediction provides an equilibrium baseline against which the experimentally measured current fluctuations can be compared.

**FIGURE 2:**
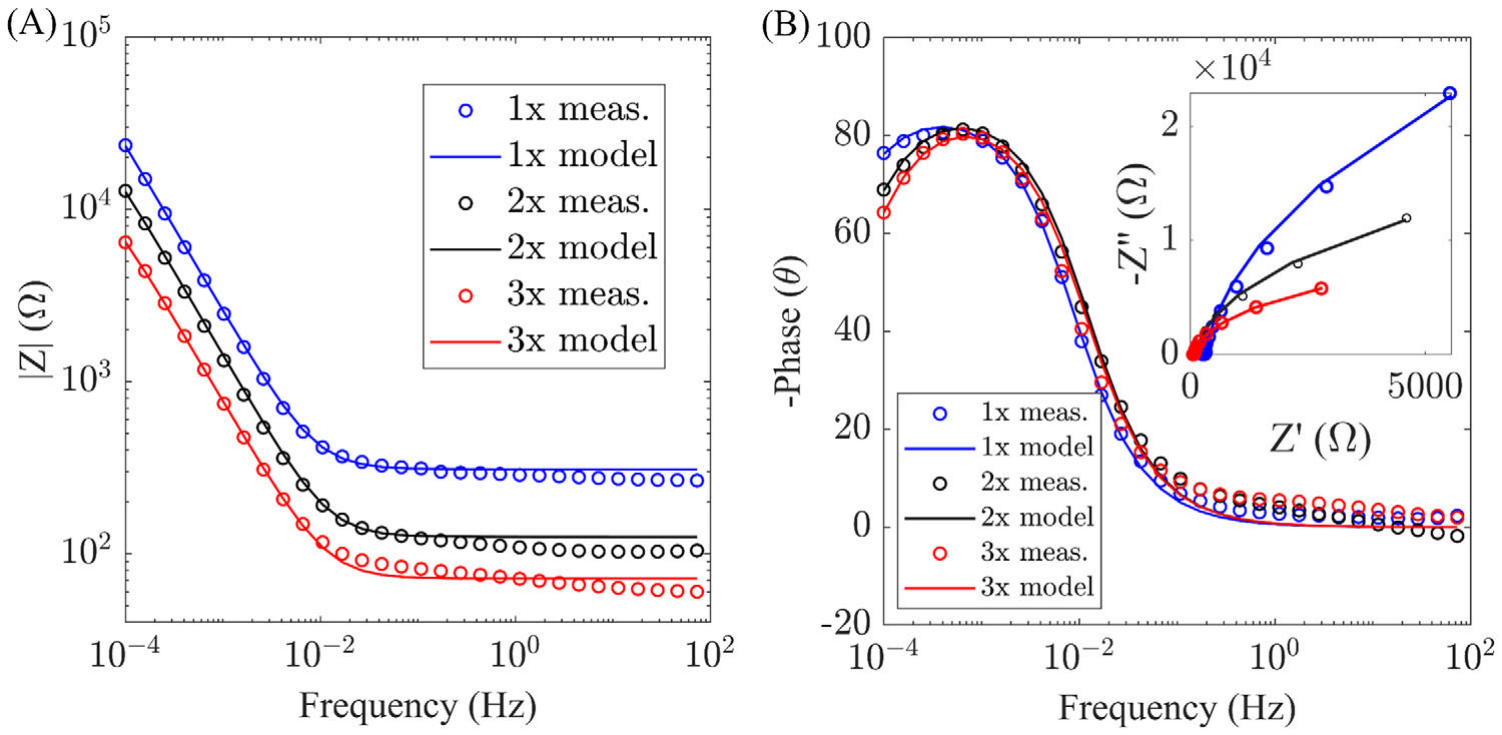
Electrochemical impedance characterization of porous PEDOT:PSS electrodes reveals low‐impedance and distributed interfacial dynamics. (A) Measured (symbols) and modelled (solid lines) impedance spectra for polyurethane foams coated once (1x), twice (2x) and three times (3x) with PEDOT:PSS. Increasing the number of dip‐coating cycles systematically reduces impedance across the frequency range 10^−4^‐10^2^ Hz, reflecting reduced series resistance and enhanced electroactive surface area. (B) Corresponding phase spectra, indicating dispersive capacitive behavior characteristic of a constant‐phase element (CPE). The three‐coat electrode exhibits a higher low‐frequency phase angle, consistent with increased effective double‐layer capacitance. The inset shows Nyquist representations (‐Z″ Vs Z′), highlighting the reduction in charge‐transfer resistance and improved interfacial kinetics with additional coating cycles. Impedance spectra were fitted over 10^−4^ − 10^2^ Hz using the *R_s_* + (*R_ct_* ∥CPE) circuit.

**TABLE 1:** Geometrical and electrochemical circuit fitting parameters (10^−4^≤ f ≤10^2 ^Hz).

Dip‐coating	PEDOT mass (mg)	Diameter (mm)	Height (mm)	*R_s_* (Ω)	*R_ct_* (kΩ)	CPE Q *(F·s^n‐1^)*	CPE n *(‐)*
1x	16.55 ± 0.27	7.48 ± 0.17	6.61 ± 0.18	309.2 ± 34.1	129.6 ± 15.6	0.054 ± 0.006	0.970 ± 0.012
2x	18.55 ± 0.32	7.31 ± 0.14	6.49 ± 0.19	125.5 ± 15.1	39.34 ± 6.72	0.100 ± 0.012	0.978 ± 0.012
3x	19.74 ± 0.23	6.92 ± 0.11	6.23 ± 0.13	71.77 ± 8.31	16.9 ± 3.03	0.178 ± 0.021	0.969 ± 0.012

**TABLE 2:** Derived volumetric and areal capacitance parameters.

Dip‐coating	*C*_*dl*,*eff*_ (mF)	*V_solid_* (cm^3^)	*A_external_* (cm^2^)	*A_internal_* (cm^2^)	*A_total_* (cm^2^)	*C_total_* (F)	*C_areal_* (mF·cm^−2^)
1x	70.34 ± 11.78	0.01655	2.432	468.37	470.80	0.3476	0.738
2x	120.47 ± 19.37	0.01855	2.330	524.97	527.29	0.3896	0.739
3x	229.98 ± 36.97	0.01974	2.107	558.64	560.75	0.4145	0.739

In the two‐electrode configuration, the equilibrium noise is determined by the total dissipative impedance of the cell, including contributions from the working electrode, solution and counter electrode. The counter electrode was fabricated from the same PU/PEDOT:PSS material as the working electrode but possessed a larger porous volume. Assuming comparable coating loading and area‐normalised interfacial properties, its effective electrochemical area scales approximately with porous volume, whereas its interfacial impedance scales inversely with that area [1]. The estimated counter‐electrode contribution should therefore be regarded as an upper bound based on the relative electrode volumes. Even when this contribution is included, the resulting correction to the equilibrium thermal‐noise floor is small relative to the several orders of magnitude excess noise measured under applied bias.

Overall, PU/PEDOT:PSS electrodes, dip‐coated 3 times (3x) yield a regime of low impedance, high capacitance and distributed electrochemical dynamics, providing the operational space required to detect small stochastic signals from electrically quiescent marine species, which we investigate in the next section through time‐resolved current measurements under applied bias.

### 2.3 Bias Induces Stochastic Current Fluctuations and Non‐Equilibrium Dynamics

We emphasize that the applied bias does not constitute a purely passive measurement, as it can influence the local electrochemical environment and charge distribution within the mixed ionic‐electronic conductor. The recorded signals should therefore be interpreted as bias‐enhanced manifestations of intrinsic stochastic processes at the diatom‐electrode interface. Importantly, the absence of large Faradaic currents and the stability of the baseline response indicate that the system operates within a weakly perturbed regime. Figure 3A shows the experimental configuration used to record current fluctuations from the porous PEDOT:PSS electrodes with and without axenic Pt15 diatoms. To establish the intrinsic electrical noise of the mesoporous PU/PEDOT:PSS electrode‐electrolyte interface, control recordings were first performed using electrolyte medium in the absence of cells. These measurements define the baseline instrumental noise against which all subsequent biological recordings are compared. The system was measured in a two‐electrode configuration, in which the porous PEDOT:PSS foam acted as the working electrode immersed in Enhanced artificial sea water medium (EASW), while a surrounding counter electrode fabricated from the same PU/PEDOT:PSS material, but with a larger porous volume and correspondingly lower interfacial impedance, completed the circuit. The WE is connected to a low‐noise transimpedance amplifier which provides both current readout and an integrated bias supply, enabling controlled DC polarization of the electrode during measurements. A constant bias voltage in the range of 0–500 mV is applied through the amplifier, driving the system into a non‐equilibrium electrochemical regime. Under applied DC bias, the system exhibits a transition from near‐equilibrium behavior to a regime dominated by stochastic current fluctuations (Figure 3B). At no bias, sporadic spikes are detected. At low bias, the current remains relatively stable, while increasing bias results in the emergence of pronounced transient events and enhanced temporal variability, indicating that the system is driven progressively out of equilibrium. The presence of living diatom populations produced stochastic electrical fluctuations substantially exceeding this baseline electrode noise, demonstrating that the excess electrical activity originates from the biological sample. The power‐law exponent (α) increased with applied bias in electrodes with axenic Pt15 diatoms, whereas only minor variations were observed for the electrolyte‐only controls (Figure 3D). Across three independent electrodes (n = 3), the mean ± SD values of α for Pt15 were 0.57 ± 0.39, 0.65 ± 0.36, 1.15 ± 0.26, 1.24 ± 0.25, 1.50 ± 0.31 and 1.17 ± 0.19 at 0, 100, 200, 300, 400 and 500 mV, respectively. The corresponding electrolyte‐only control values were 0.67 ± 0.48, 0.65 ± 0.19, 0.49 ± 0.25, 0.81 ± 0.04, 0.66 ± 0.03 and 0.78 ± 0.04.

**FIGURE 3:**
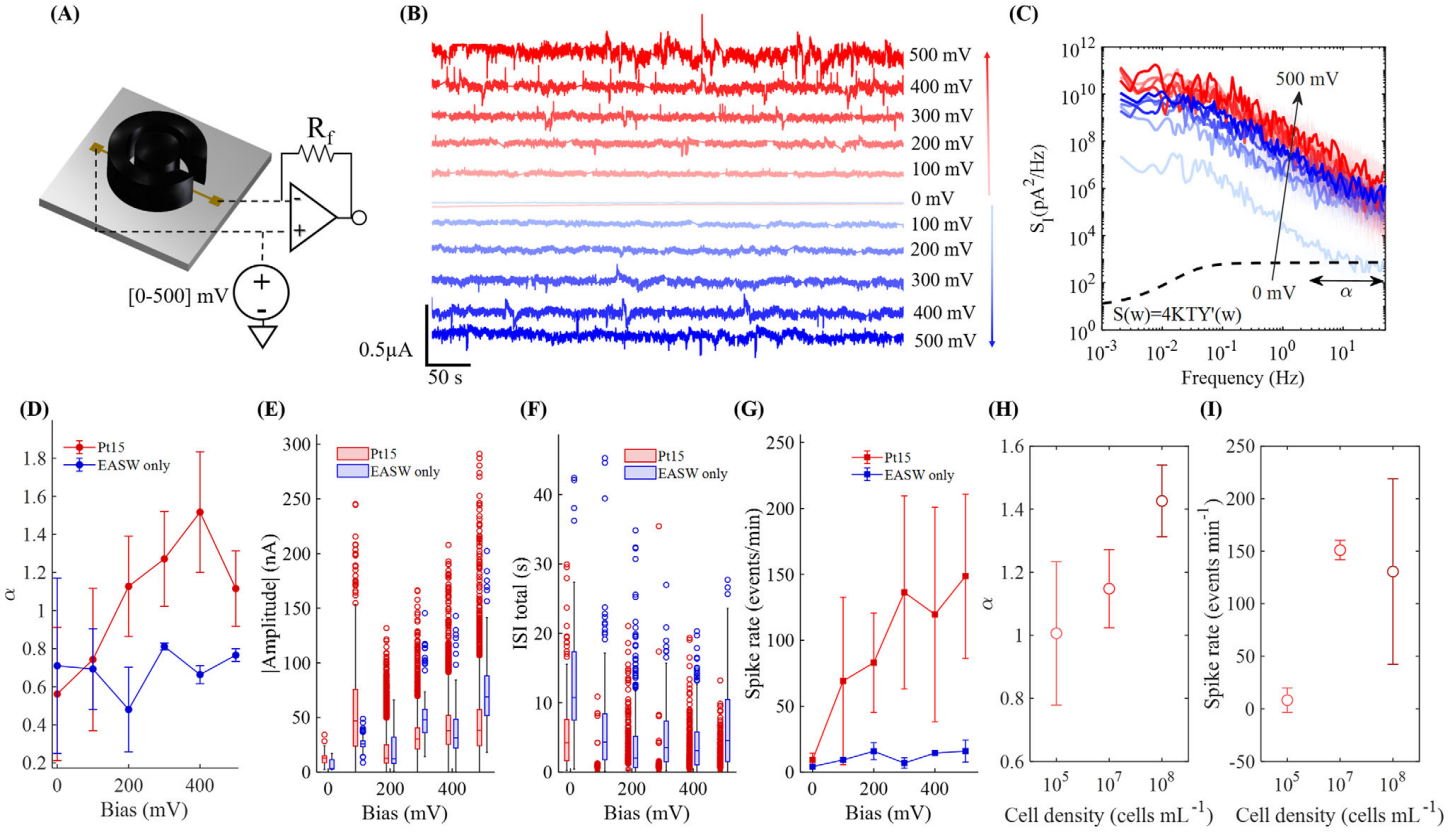
Bias‐dependent stochastic electrical fluctuations and cell‐density sensing in axenic *Phaeodactylum tricornutum* interfaced with porous PU/PEDOT:PSS electrodes. (A) Schematic of the electrical measurement configuration. A porous PU/PEDOT:PEDOT:PSS working electrode interfaced with electrolyte is connected to a low‐noise transimpedance amplifier (feedback resistor, R_f_) while a DC bias (0–500 mV) is applied between the working and counter electrodes. (B) Representative current traces recorded from electrodes containing axenic Pt15 cultures (red) and electrolyte‐only controls (blue) under increasing DC bias. (C) Current noise spectra from 1 to 50 Hz, shown as thin traces (individual measurements) and thick lines (log‐binned median). The dashed line represents the theoretical thermal noise floor derived from electrochemical impedance spectroscopy (EIS) using Equation (3). (D) Bias dependence of the power‐law exponent α extracted from fits to *S*_*I*_(*f*)∝*f* ^−*α*^. Power spectral densities were calculated over the 10^−3^–50 Hz frequency range, while *α>* was determined by fitting the PSD over the 1–50 Hz interval used for quantitative analysis. (E) Distribution of transient current amplitudes as a function of applied bias. (F) Distribution of inter‐spike intervals (ISI) as a function of applied bias. (G) Spike rate (events min^−1^) as a function of applied bias for Pt15 electrodes and electrolyte‐only controls. (H) Dependence of the PSD power‐law exponent α on Pt15 cell density (10^5^, 10^7^ and 10^8^ cells mL^−1^) measured at 500 mV. Symbols represent the mean and error bars denote the standard deviation across independent electrodes. (I) Dependence of spike rate on Pt15 cell density measured at 500 mV. Unless otherwise stated, all measurements were performed in artificial seawater (EASW), and error bars represent the standard deviation from independent electrodes (*n* = 2–3).

Impedance spectra were also recorded and modelled under polarization ranging from 0–500 mV (Figure S5). Across the investigated bias range, the constant phase element (CPE) exponent remains close to unity (*n* ≈ 0.92 − 0.96), indicating that the electrode‐electrolyte interface retains a predominantly capacitive character despite significant polarization. In contrast, the charge‐transfer resistance *R_ct_* increases systematically with increasing bias. This behavior reflects the strong influence of DC polarization on the electrochemical processes occurring within the porous mixed ionic‐electronic network. In PEDOT:PSS electrodes, the applied bias modifies the electrochemical doping state of the polymer backbone and drives redistribution of mobile ions within the polymer network. Such ionic rearrangements alter the local electrostatic environment and modulate the effective charge‐transfer kinetics at the electrode‐electrolyte interface. Additional contributions may arise from polarization of the electrolyte within the porous scaffold and from bias‐induced changes in the accessibility of electrochemically active sites.

The impedance analysis at different bias (Figure S5) indicates that the porous PEDOT:PSS interface exhibits distributed electrochemical dynamics instead of a single well‐defined relaxation process. In porous mixed ionic‐electronic conductors, microscopic variations in pore geometry, ionic accessibility and local electrochemical potential generate a broad distribution of relaxation times associated with ionic polarization, interfacial charge transfer and ion transport within the polymer network. The temperature‐dependent measurements (Figure S6) further support this interpretation, showing that the low‐frequency response evolves systematically with temperature, consistent with thermally activated ionic redistribution within the porous structure. Such distributed interfacial dynamics naturally give rise to dispersive impedance behavior and provide a physical basis for the low‐frequency stochastic fluctuations observed under DC polarization in the following sections. The temperature‐dependent impedance measurements provide additional evidence for the distributed nature of the electrochemical dynamics in the porous PEDOT:PSS electrodes. In particular, the impedance modulus exhibits a reproducible deviation from the dominant constant‐phase behavior in the low‐frequency region around 1 Hz. This feature appears as a broad shoulder in ∣Z∣ and becomes clearer when analysing the loss tangent tan(α) = ‐Z″/Z′. Unlike an ideal RC relaxation, which would produce a sharp peak in tan(α) at a well‐defined frequency, the present response remains broad and dispersive across temperature. This behavior is consistent with a distribution of relaxation processes. The gradual temperature dependence of tan(α) evaluated at a fixed reference frequency (0.4 Hz) further indicates that the underlying process is thermally activated and likely associated with ionic transport and polarization within the PEDOT:PSS network. Importantly, this low‐frequency relaxation occurs within the frequency window where enhanced stochastic current fluctuations are observed under DC bias, suggesting that the distributed ionic‐electronic polarization processes identified by impedance spectroscopy provide the physical origin of the low‐frequency electrical noise analysed in the following sections. The distributed relaxation behavior inferred from impedance spectroscopy therefore provides a physical origin for the low‐frequency electrical fluctuations observed under DC polarization, since stochastic variations in ionic transport and interfacial charge‐transfer processes can modulate the effective conductance of the electrode‐electrolyte interface. The stochastic ionic and electrochemical processes within the porous mixed ionic‐electronic interface provide the fundamental source of the current noise analysed in the following sections. This connection between distributed electrochemical relaxation and stochastic current fluctuations provides a consistent physical framework linking the impedance spectroscopy and noise measurements presented in this work.

Importantly, the measured noise spectra exceed the Johnson‐Nyquist thermal noise level estimated from the impedance‐derived admittance by several orders of magnitude (Figure 3C). At 0 mV, the measured current noise spectrum approaches the thermal‐noise expectation over most of the accessible bandwidth, indicating that the system operates close to equilibrium under these conditions. The deviation observed below 1 Hz is attributed to the increased influence of ultra‐slow baseline fluctuations. Such low‐frequency features originating from slow drifts, environmental fluctuations and detrending effects, although common in finite‐time measurements, are not captured by the ideal Johnson‐Nyquist description. Because the noise measurements were performed in a two‐electrode configuration whereas the impedance characterization primarily describes the working electrode interface, the calculated Johnson‐Nyquist spectrum should be regarded as a conservative estimate of the equilibrium noise floor. Owing to the substantially lower impedance of the large‐area counter electrode, its contribution to the total dissipative impedance is expected to be comparatively small and does not alter the conclusion that the experimentally measured spectra exceed the equilibrium prediction by several orders of magnitude. Even under this conservative interpretation, the experimentally measured noise spectra remain several orders of magnitude higher than the equilibrium thermal‐noise prediction. This substantial excess noise cannot be accounted for by Johnson–Nyquist fluctuations alone, therefore indicating the presence of non‐equilibrium interfacial processes. These fluctuations can be understood as arising from time‐dependent variations in the effective interfacial conductance, driven by ionic motion, interfacial polarization and charge redistribution within the porous mixed‐conducting network. The volumetric nature of the electrode enables the superposition of many such local processes, giving rise to the observed stochastic behavior.

To verify that the electrical stimulation protocol did not induce widespread membrane damage, additional confocal fluorescence imaging was performed following continuous exposure to the different bias conditions for 4 h. As shown in Figure S10, dense populations of *P. tricornutum* remained attached throughout the porous PU/PEDOT:PSS scaffold under all stimulation conditions. Only occasional SYTOX^TM^ Green‐positive cells were observed, with no qualitative increase following prolonged exposure to 500 mV. These observations indicate that the applied electrical stimulation preserves the overall membrane integrity of the diatom population.

Furthermore, to investigate whether the stochastic electrical fingerprints also encode microalgal population density, additional measurements were performed using three independent *P. tricornutum* concentrations (10^5^, 10^7^ and 10^8^ cells mL^−1^) under identical experimental conditions. Representative analyses acquired at 500 mV are summarized in Figure 3H,I. The spectral exponent α increased progressively with increasing cell density, indicating that higher cell populations generate increasingly correlated low‐frequency current fluctuations within the porous PU/PEDOT:PSS interface. In contrast, the spike rate increased markedly from 10^5^ to 10^7^ cells mL^−1^ but exhibited only a modest further increase at 10^8^ cells mL^−1^. This behavior suggests that, although increasing biomass continues to strengthen the collective fluctuation dynamics captured by the power spectral density, the number of individually resolved stochastic events approaches saturation at high cell densities. To deepen this further, we employ scanning electron microscopy (Figure S9). As cell density increases, progressively larger fractions of the porous electrode become occupied by adhered *P. tricornutum* cells, resulting in extensive surface coverage at 10^8^ cells mL^−1^. Under these conditions, neighbouring cells are likely to share conductive pathways and produce temporally overlapping current fluctuations, thereby reducing the number of individually distinguishable transient events while maintaining increasingly correlated collective dynamics. These observations demonstrate that stochastic electrochemical noise spectroscopy provides complementary frequency‐domain and time‐domain descriptors that are sensitive to microalgal population density and further support its potential as a label‐free bioelectronic sensing strategy.

To further provide biological context for the density‐dependent stochastic electrochemical measurements, the growth behavior of the axenic Pt15 culture was independently monitored as shown in Figure S8. The culture followed the expected logistic growth profile, reaching a stationary population of approximately 2 × 10^6^ *cells* *mL* ^−1^ after 10–14 days. The cell densities investigated in Figure 3H,I therefore span more than three orders of magnitude relative to the natural culture trajectory, enabling the influence of microbial population density on stochastic electrical fluctuations to be evaluated independently of the intrinsic batch growth dynamics.

To further evaluate whether the enhanced stochastic signals originated from the three‐dimensional mesoporous architecture, we compared the recordings with planar PEDOT:PSS electrodes fabricated from the same conducting polymer and measured under identical biological conditions. After normalisation to architecture‐specific media controls, mesoporous PU/PEDOT:PSS electrodes continued to exhibit higher event rates, greater transient prominence and larger integrated low‐frequency PSD than planar PEDOT:PSS electrodes (Figure S7), suggesting that the observed enhancement reflects improved detection of biologically generated electrical fluctuations arising from the increased electroactive surface area and improved cell‐electrode coupling afforded by the mesoporous architecture. While planar microelectrodes can offer advantages in spatial resolution by sampling smaller extracellular volumes and may better resolve individual electrical events, the present results indicate that for population‐level recordings of *P. tricornutum*, the three‐dimensional mesoporous architecture provides superior sensitivity to stochastic bioelectrical activity.

### 2.4 Pharmacological Modulation of Spontaneous Bioelectrical Activity by TEA

To investigate whether the observed stochastic bioelectrical activity is associated with membrane ion‐channel function, we examined the effect of tetraethylammonium (TEA), a classical pharmacological inhibitor of many TEA‐sensitive potassium channels in both animal and plant systems and one of the most widely used probes of voltage‐dependent K^+^ conductance [3, 20, 21, 22, 23]. Before performing electrophysiological measurements, we first verified that TEA produced a measurable physiological response in *P. tricornutum* under the experimental conditions used for electrical recordings. Because potassium transport contributes to the regulation of photosynthetic energy dissipation and thylakoid membrane function in photosynthetic organisms, alterations in non‐photochemical quenching (NPQ) provide a sensitive physiological readout of membrane perturbation [24] while preserving overall photosynthetic competence. Pulse‐amplitude‐modulated chlorophyll fluorescence measurements showed that exposure to 10 mm TEA for 15 min reduced NPQ over the entire irradiance range (Figure 4A). The decrease became progressively more pronounced with increasing actinic irradiance, where NPQ normally reaches its highest values, indicating that TEA impaired the cells capacity to dissipate excess excitation energy. Complementary measurements of F_0_, Fv/Fm and ETR_max_ showed no substantial changes under these conditions (not shown), demonstrating that the treatment did not produce major damage to PSII photochemistry or compromise overall photosynthetic performance. These observations indicate that TEA maintained cells viable and metabolically active, validating the use of these conditions for pharmacological interrogation of extracellular electrical activity.

**FIGURE 4:**
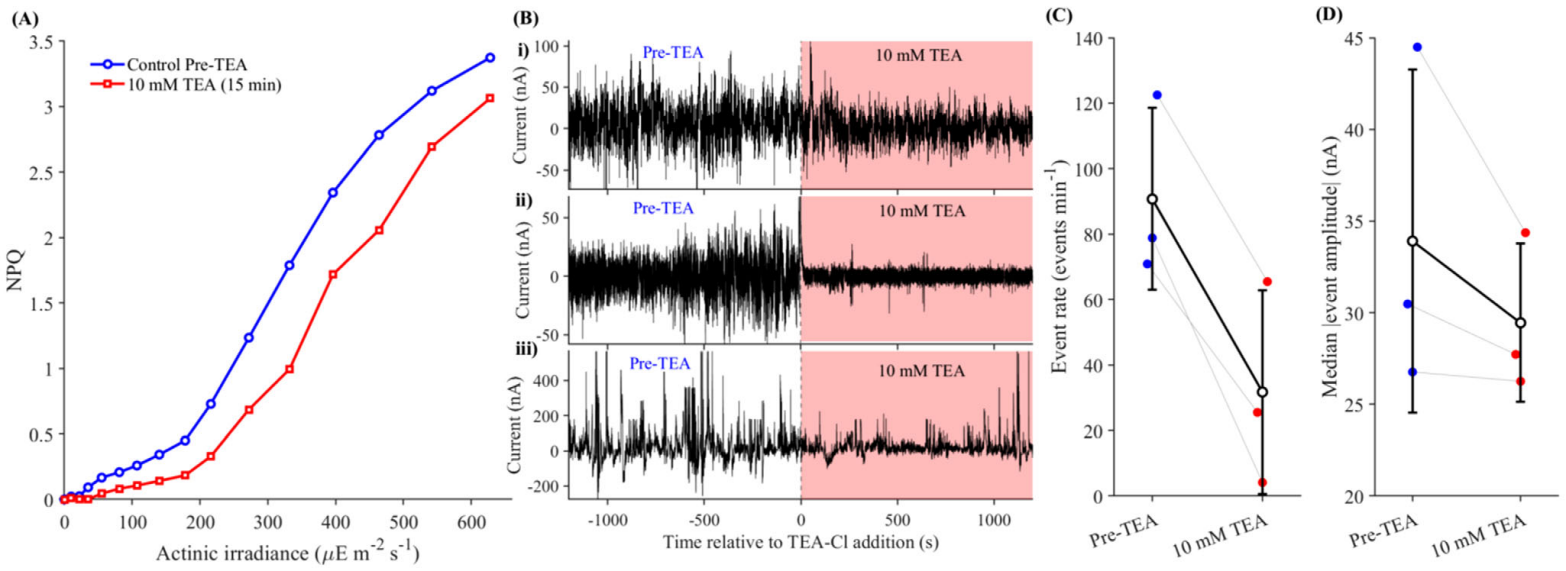
Pharmacological modulation of physiological and spontaneous bioelectrical activity by tetraethylammonium (TEA). (A) Effect of 10 mM tetraethylammonium (TEA) on non‐photochemical quenching (NPQ) in *Phaeodactylum tricornutum* showing preserved photosynthetic activity. (B) Representative current recordings obtained from three independent Pt15 biofilms before (Pre‐TEA) and after addition of 10 mm TEA. The vertical dashed line denotes the time of TEA addition. Shaded regions indicate the analysis interval after (red) pharmacological treatment. (C) Paired quantification of spontaneous transient‐event frequency before and after TEA treatment (n = 3 independent biological replicates). Colored symbols represent individual experiments, and black symbols indicate the mean ± SD. (D) Paired quantification of the median transient‐event amplitude from the same recordings shown in (C). TEA decreased both the frequency and amplitude of spontaneous extracellular bioelectrical transients, consistent with pharmacological modulation of potassium‐dependent membrane processes.

Having established that TEA effectively modulated cellular physiology, we next examined its effect on spontaneous extracellular electrical dynamics. Current recordings revealed a clear attenuation of stochastic fluctuations immediately following TEA addition (Figure 4B). Across independent biological replicates (n = 3), TEA consistently reduced the frequency of spontaneous transient electrical events (Figure 4C), accompanied by a modest decrease in their median amplitude (Figure 4D), further demonstrating that spontaneous extracellular electrical activity in *P. tricornutum* is pharmacologically modulated by TEA‐sensitive membrane processes. Although TEA is not selective for a single potassium‐channel subtype and additional membrane transport systems may contribute to the observed response, these experiments provide independent functional evidence that potassium‐dependent membrane processes participate in generating the measured stochastic bioelectrical dynamics. Future studies combining complementary pharmacological probes, targeted genetic perturbation of candidate ion transporters, and simultaneous electrophysiological and optical measurements will be required to identify the underlying molecular mechanisms.

### 2.5 Axenic Diatoms Modulate Interfacial Transport and Electrochemical Noise

To reduce ambiguity associated with mixed microbial communities, all experiments were performed using axenic cultures of *P. tricornutum*. In non‐axenic systems, extracellular electrical signals may arise from a combination of diatom activity, bacterial metabolism and interspecies interactions, including the production of redox‐active metabolites and biofilm‐mediated charge transfer. By employing axenic cultures, we constrain the origin of the measured electrical signals to diatom‐associated processes, thereby enabling a more direct interpretation of the coupling between cellular activity and the bioelectronic interface. This establishes stochastic electrochemical signals as an informative probe of dynamic bioelectrochemical processes in microbial systems.

Axenic diatoms adherent to PU/PEDOT:PSS electrodes modify interfacial transport and enhance stochastic electrochemical fluctuations. In the presence of axenic cultures of Pt15, the stochastic current fluctuations are significantly enhanced compared to electrolyte‐only controls (Figure 3D–G). This enhancement is most clearly reflected in an increased frequency of transient stochastic events (Figure 3G). In addition, the event‐amplitude distributions show a modest upward shift and broader dispersion in the presence of cells (Figure 3E), although this effect is less pronounced than the change in event rate, suggesting that applied bias primarily amplifies the electrical readout of biologically modulated stochastic interfacial dynamics. The biological modulation of interfacial transport can be further deepened with cyclic voltammetry. Figure 5 shows the scan‐rate dependent cyclic voltammetry response of this electrode in electrolyte‐only conditions (EASW) and after 11 days of incubation with axenic Pt15 cultures. In both conditions, broad hysteretic CV loops are retained across the full scan‐rate range, suggesting preserved volumetric electrochemical activity of the porous mixed ionic‐electronic conducting scaffold. Such behavior is characteristic of porous PEDOT:PSS systems in which charge storage arises from distributed ionic‐electronic coupling throughout the bulk [25].

**FIGURE 5:**
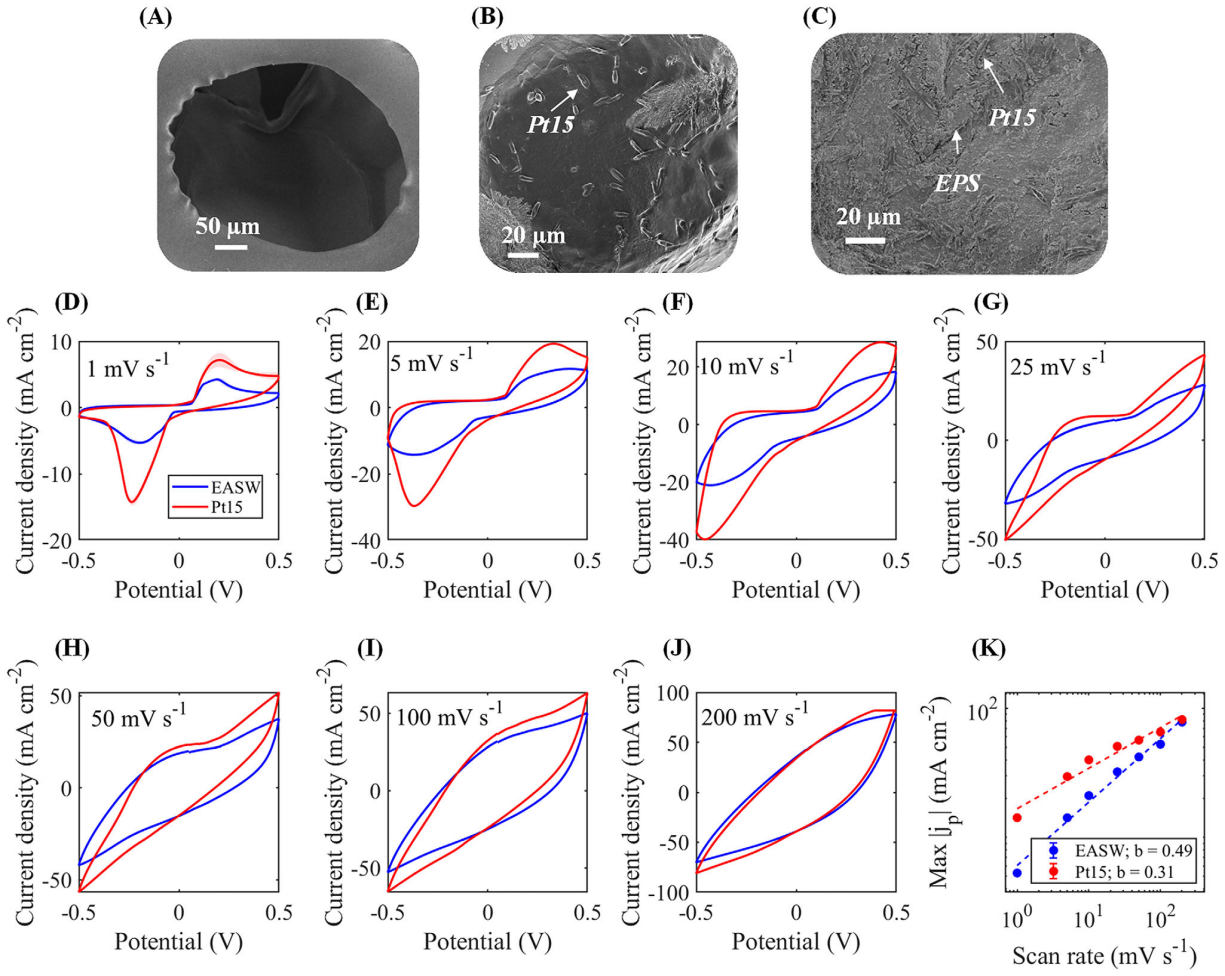
Morphology and scan‐rate dependent cyclic voltammetry of the porous PU/PEDOT:PSS electrode before and after seeding axenic Pt15 cells. (A‐C) Scanning electron microscopy (SEM) images of (A) bare PU/PEDOT:PSS electrodes highlighting the porous surface morphology, (B) Pt15 cells adherent on the porous electrode taken at the end of the bias experiment, demonstrating intimate physical integration between Pt15 and the conductive scaffold and (C) fibrillar extracellular material, likely extracellular polymeric substances (EPS), on the PEDOT:PSS surface, increasing interfacial roughness and ionic interaction area (scale bar 20 µm). (D‐J) Scan‐rate‐dependent cyclic voltammetry of mesoporous PU/PEDOT:PSS electrodes in the presence and absence of axenic Pt15 diatoms. Representative cyclic voltammograms obtained from one independent electrode, are shown for EASW‐only controls (blue) and electrodes containing axenic Pt15 cells (red) at scan rates of 1, 5, 10, 25, 50, 100 and 200 mV s^−1^. For each condition and scan rate, the solid line represents the mean of scans 2–4, while the shaded region represents the corresponding standard deviation across these sequential scans. Scan 1 was excluded as a conditioning cycle. Current was normalised to the projected geometric electrode area of 0.376 cm^2^. The final panel shows the scan‐rate dependence of the maximum absolute peak current density, fitted according to *j_p_* =  *av^b^*. The fitted exponents were b = 0.49 for EASW and b = 0.31 for Pt15. Across three independent electrodes, the mean ± SD values were b = 0.521±0.032 for EASW and b = 0.362±0.042 for Pt15 (*n* = 3). The lower b‐value in the presence of cells indicates a weaker scan‐rate dependence and an increased contribution from slow, transport‐limited ionic and interfacial processes at the porous bioelectrode interface.

At low scan rates, Pt15‐colonized electrodes exhibit clear branch‐dependent deviations from electrolyte‐only controls, including a more pronounced cathodic response at 1 mV s^−1^ and redistribution of the anodic current at 5–10 mV s^−1^. These differences emerge on longer timescales, when ions can access a larger fraction of the porous network, indicating that the biofilm modifies slow ionic transport and interfacial charging within the scaffold. Consistent with this interpretation, the peak current density followed the power‐law relationship *j_p_* =  *av^b^*, with the fitted exponent decreasing from b = 0.521±0.032 for electrolyte‐only controls to b = 0.362±0.042 after Pt15 colonization across three independent electrodes (n = 3). The lower exponent indicates a weaker dependence of peak current on scan rate and is consistent with an increased contribution from distributed, transport‐limited processes within the biofilm‐coated porous electrode. Such behavior may arise from restricted ionic motion, heterogeneous accessibility of the electrochemically active surface, finite‐length diffusion, and distributed resistive‐capacitive pathways within the tortuous PEDOT:PSS network [25, 26, 27, 28, 29]. At higher scan rates, the Pt15 and electrolyte‐only voltammograms overlap more closely, indicating that the faster charging response is comparatively less affected. This scan‐rate‐dependent behavior differs from classical blocking by an insulating layer, which would be expected to suppress current more broadly across the investigated timescales [11, 12, 13]. Thus, both CV analysis and SEM observations of abundant extracellular polymeric material surrounding the immobilized diatoms, support the hypothesis that the biofilm matrix modifies ionic transport within the porous bioelectrode while preserving its volumetric electrochemical activity.

Electrochemical impedance spectroscopy during biofilm formation provides additional mechanistic insight. While the high‐frequency response remains similar between conditions, Pt15 exhibits a pronounced increase in mid‐frequency impedance and a distinct phase maximum not observed in the electrolyte‐only control. The emergence of such features is characteristic of additional or shifted relaxation processes in distributed systems, often associated with changes in ionic transport pathways or dielectric properties within porous networks [15, 30, 31]. Because microscopy revealed extracellular polymeric substances (EPS) partially covering the pore walls (Figure 5C), the most plausible interpretation is that EPS and cellular material partially occupy the porous architecture, increasing ionic tortuosity and modifying local ion‐polymer interactions without disrupting electronic percolation. Hence, Pt15 biofilm formation induces a reorganization of distributed charging dynamics, primarily affecting intermediate and slow timescales. We further deepened the impedance analysis using a transmission‐line model [26, 27] which captures the distributed coupling between ionic transport in the electrolyte‐filled pores and electronic conduction in the polymer backbone (see Figure S11 and derived circuit theory in Section 4.7). In media‐only and after biofilm formation, the leakage resistance (*R_leak_*) converges to values exceeding 10^10^ *Ω*, indicating that the data does not require a distributed conductive leakage pathway. Instead, the primary differences are captured by changes in the pore admittance (*Q_p_*, *n_p_*), the distributed resistance (*r_t_*) and the terminal branch (*R_t_*,*Q_t_*). Notably, biofilm formation leads to an increase in *Q_p_* and *r_t_* together with a decrease in the effective terminal resistance *R_t_*. Within the finite‐length transmission‐line model, *R_t_* represents the effective electrochemical termination impedance of the distributed porous network. Accordingly, the observed reduction in *R_t_* should be interpreted as a redistribution of ionic transport pathways and electrochemical relaxation processes arising from partial occupation of the porous architecture by cells and extracellular polymeric substances. Therefore, the transmission‐line parameters should be interpreted as effective descriptors of the collective electrochemical behavior of the biohybrid interface. While the finite‐length transmission‐line model captures the dominant distributed transport processes within the porous PEDOT:PSS electrode, it does not explicitly describe the spatial heterogeneity introduced by individual cells or extracellular polymeric substances.

The emergence of an additional relaxation process suggests that biological material, likely EPS covering the pore walls, modulates ion transport and charge storage across multiple length scales within the electrode. Furthermore, the impedance magnitude response (Figure S11A) reveals a clear divergence between electrolyte‐only and colonized conditions in the intermediate‐to‐low frequency regime (10^−2^–10^3^ Hz), while the high‐frequency response remains comparable. This indicates that bulk electrolyte resistance and electronic conduction through the scaffold are largely preserved and that the dominant biological effect arises from modifications to distributed interfacial and pore‐mediated processes. The phase response (Figure S11B) provides more direct insight into the underlying mechanisms. The electrolyte‐only condition exhibits a single broad relaxation associated with distributed capacitive charging of the porous network. In contrast, the colonized electrode displays a pronounced additional phase feature in the mid‐frequency range, indicative of a second characteristic timescale. This additional relaxation is consistent with a restructuring of ionic transport pathways within the porous architecture, particularly with partial filling of the porous network by cells and EPS, which increase ionic path length, modify local conductivity and introduce additional heterogeneity. Thus, biofilm formation does not suppress electrochemical activity but instead reconfigures the distributed transport landscape of the system.

The power spectral densities exhibit a characteristic power‐law dependence *S_I_* (*f*)∝*f* ^−α^ over the analysed frequency range (Figure 3C,D,I), with spectral exponents increasing from near‐thermal values at low bias to substantially larger low‐frequency scaling exponents under stronger polarization, indicating broadband non‐equilibrium stochastic dynamics at the electrode‐electrolyte interface. This behavior is consistent with the distributed interfacial dynamics identified by impedance spectroscopy (Figure S5). Importantly, except when no bias is applied e.g., in thermodynamic equilibrium, the measured noise spectra exceed the Johnson–Nyquist thermal noise predicted from the impedance‐derived admittance by several orders of magnitude across the entire bias range. This observation demonstrates that the interface under DC polarization operates in a strongly non‐equilibrium regime. While excess noise is present in both media‐only and diatom‐containing systems, the presence of diatoms consistently increases the amplitude of the noise spectra and enhances the low‐frequency components. This indicates that biological processes modify the stochastic electrochemical dynamics occurring at the electrode interface.

### 2.6 Biological Modulation of Stochastic Interfacial Dynamics

The current traces become increasingly pronounced as the applied bias increases, suggesting that biological processes associated with the diatoms introduce additional stochastic activity at the electrode interface. Possible mechanisms for these transient events include fluctuations in ion transport across cell membranes, localized electrochemical reactions near the cell surface or dynamic changes in the ionic microenvironment caused by cellular metabolism. While the present data do not uniquely identify the microscopic origin of these events, they demonstrate that the porous PEDOT:PSS electrodes can resolve stochastic electrical activity associated with living microorganisms.

The enhanced fluctuations observed in the presence of diatoms therefore indicate that biological processes introduce additional stochastic ionic dynamics within the porous electrode microenvironment. Even in the absence of biological activity, electrochemical interfaces under bias are known to generate low‐frequency noise arising from stochastic interfacial processes. In electrolyte‐only control experiments, under bias, the measured current spectra exhibit a characteristic power‐law form of 1/f. Such spectra are widely observed in electrochemical systems and are generally attributed to the superposition of many relaxation processes with broadly distributed characteristic times. Several mechanisms may contribute to this behavior at the porous PEDOT:PSS interface. First, stochastic adsorption and desorption of ions or redox‐active species can modulate the local electrochemical potential at the electrode surface [32]. Second, charge‐transfer reactions occurring at discrete active sites may exhibit intermittency due to fluctuations in local ionic concentration or surface states [33, 34]. Third, in mixed ionic‐electronic conductors such as PEDOT:PSS, applied bias can drive slow ionic migration within the polymer matrix, dynamically modifying the local doping state and electrostatic environment of the conducting polymer backbone [35, 36].

In porous architectures, these ionic redistribution processes can occur over a wide range of spatiotemporal scales. The resulting ensemble of relaxation processes naturally produces low‐frequency noise with 1/*f* spectral dependence. The observed increase in both fluctuation amplitude and spectral exponent with applied bias is consistent with a bias‐driven shift in the balance between capacitive charge storage, interfacial polarization and faradaic exchange processes. The additional stochastic activity observed in the presence of diatoms may arise from biological ion‐transport processes occurring at the cell membrane. Many marine microalgae possess membrane transport systems, including voltage‐sensitive ion channels, proton pumps and other electrogenic transporters that dynamically regulate intracellular ion concentrations and membrane potential [37, 38]. Perturbations of the extracellular electrochemical environment caused by the applied bias may therefore influence membrane transport activity and lead to stochastic ionic fluxes in the vicinity of the electrode. Such fluctuations could modulate the local ionic composition within the porous PEDOT:PSS network and thereby contribute to the enhanced current noise observed in the biological experiments. Although the present measurements do not directly identify the underlying biological mechanism, membrane‐associated ion‐transport processes provide a plausible source of the additional stochastic fluctuations observed in the presence of diatoms.

Metabolic activity may also influence the observed fluctuations by modifying local ionic composition, redox conditions or pH in the vicinity of the electrode. Such chemical perturbations can alter ion transport within the PEDOT:PSS network and influence interfacial charge‐transfer processes. Although the present experiments do not uniquely identify the microscopic biological mechanism, the systematic difference between media‐only controls and diatom‐containing systems demonstrates that living cells introduce additional stochastic electrochemical dynamics. Overall, these results demonstrate that porous PEDOT:PSS electrodes enable sensitive detection of microalgae‐associated stochastic current transients and excess interfacial noise above the abiotic background signals. Pt15 biofilm modulates stochastic interfacial conductance through fluctuations in local ionic concentration and electrochemical potential within the porous network. In this framework, the Pt15 biofilm dynamically perturbs the distributed ionic‐electronic transport pathways, leading to time‐dependent variations in effective admittance that manifest as excess current noise.

### 2.7 Stochastic Conductance and Diffusion‐Limited Ionic Coupling in Pt15 Populations

The stochastic electrical fluctuations observed in this work emerge as excess low‐frequency noise superimposed on the thermal baseline of the electrochemical system and are therefore most naturally interpreted as fluctuations in interfacial conductance. In mixed ionic‐electronic systems such as PEDOT:PSS, the measured current reflects the coupling between electronic transport in the polymer and ionic dynamics in the electrolyte. Accordingly, temporal variations in local ionic concentration, interfacial polarization and charge distribution can give rise to measurable conductance fluctuations, which are transduced into current noise under applied bias. Within this framework, the observed noise should reflect the superposition of many microscopic processes with distributed time scales. These include stochastic ion transport across cellular membranes, adsorption and desorption processes at the electrode interface and fluctuations in the local ionic environment surrounding the cells. Such processes are expected to generate broad‐band noise spectra with enhanced low‐frequency components, consistent with the experimentally observed behavior. At sufficiently strong non‐equilibrium bias, diffusion‐like stochastic transport can also produce spectral exponents approaching 3/2, as previously reported in other bias‐driven systems, reinforcing the interpretation that part of the observed excess noise may arise from slow diffusion‐mediated fluctuations instead of classical 1/f conductance noise [2, 4, 37]. Fluctuations in the concentration of ions or signalling molecules, arising from secretion, uptake or local metabolic activity, can propagate through the extracellular medium and modulate the interfacial impedance over time. This diffusion‐limited transport introduces long correlation times and spatial coupling between neighbouring cells, providing a physical basis for collective, yet unsynchronized, electrical fluctuations within dense Pt15 populations. The applied bias enhances the electrical readout of these processes, enabling their detection against the intrinsic noise floor of the system.

Diatoms are known to possess a range of ion transport mechanisms, including voltage‐sensitive and ligand‐gated channels, which regulate intracellular ion homeostasis and signalling. While these processes have primarily been studied using genetic and optical approaches, their electrical signatures at the population‐electrode interface remain poorly understood. In this context, the excess noise observed in Pt15‐electrodes can be interpreted as arising from stochastic modulation of interfacial conductance driven by ionic fluxes. The genome of *P. tricornutum* encodes a diverse repertoire of putative voltage‐gated ion channels, consistent with the capacity of diatoms to couple environmental and electrochemical stimuli to rapid ionic responses [10, 32, 33]. The Pt15 accession used in this study has been genomically characterized and its annotation identifies multiple candidate ion‐transport and voltage‐gated channel proteins (Table S4), providing a plausible molecular basis for electrically and chemically responsive membrane behavior [39, 40]. Although only a subset of these channels has been functionally characterized experimentally in other accessions, their collective presence provides a plausible molecular basis for dynamic ionic fluctuations within diatom populations, capable of modulating the local electrochemical environment [10].

The present measurements are consistent with biologically driven fluctuations in local ionic concentration, interfacial polarization and electrochemical conditions at the Pt15‐electrode interface. Importantly, our data do not directly resolve single ion channels and do not demonstrate globally synchronized action‐potential‐like events across the entire diatom population. Our data shows that living axenic Pt15 cells introduce excess stochastic admittance fluctuations that are absent or much weaker in media‐only controls and that these biological fluctuations become more experimentally evident under applied bias because current fluctuations scale with conductance fluctuations according to δ*I*(*t*)  =  *V* δ*G*(*t*)  [41, 42]. Thus, weak cell‐associated signals can be detectable even at zero bias, but DC polarization amplifies their electrical readout and increases the contrast relative to the abiotic electrochemical background.

One possible conceptual framework for interpreting these observations is diffusion‐limited paracrine communication within local cell cohorts. As discussed previously for microalgal systems, if a signalling ion released by one cell diffuses with diffusion coefficient D and persists for a characteristic lifetime τ_*p*_ before uptake or recombination, the associated diffusion length can be expressed as $L_{p}=\sqrt{D\tau_{p}}$ [3]. In dense local populations, this defines a handover distance over which extracellular ionic perturbations can influence neighbouring cells. We therefore suggest that the excess noise and altered event statistics are consistent with bias‐assisted detection of local, stochastic and diffusion‐limited ionic signalling within diatom cohorts. The porous PEDOT:PSS scaffold does not simply measure steady‐state current, and instead it transduces transient fluctuations in local ionic concentration, interfacial polarization and cell‐associated conductance into measurable stochastic electrical signals. The present data support biologically modulated, diffusion‐mediated and bias‐amplified stochastic electrical activity, but they do not yet ascertain long‐range synchronization or direct cell‐to‐cell ion exchange with single‐cell resolution. Future work combining pharmacology, genetically informed perturbation of ion‐transport pathways and spatially resolved electrical or optical readouts will be required to determine whether the detected fluctuations arise from ion‐channel‐mediated paracrine signalling, coordinated subpopulation dynamics or even both.

### 2.8 Implications for Non‐Equilibrium Measurements and Microbial Ecophysiology

The bias dependence of the current noise spectra and the large excess above the equilibrium thermal noise predicted from impedance spectroscopy indicate that the porous PU/PEDOT:PSS electrode‐electrolyte interface operates in a non‐equilibrium regime under DC polarization. Within this fluctuating electrochemical system, the presence of living diatoms introduces additional stochastic dynamics that amplify the observed current noise and modify the statistics of transient events. The porous PEDOT:PSS electrode therefore functions as a sensitive interface capable of transducing complex electrochemical and biological fluctuations occurring at the microbe‐electrode interface. Once stochastic fluctuations integrate contributions from a wide range of microscopic processes, noise spectra analysis provides a complementary window into interfacial dynamics that are often inaccessible through conventional steady‐state measurements. The porous PEDOT:PSS architecture is particularly advantageous in this context because its mixed ionic‐electronic conducting network enables volumetric coupling between ionic processes in the electrolyte and electronic readout through the conducting polymer backbone. This coupling reduces interfacial impedance while simultaneously increasing the effective electrochemical interaction volume, thereby enhancing sensitivity to weak stochastic fluctuations.

Beyond the electrochemical interface itself, these findings suggest new opportunities for electrical interrogation of microbial systems in which weak, distributed and time‐dependent ionic processes are otherwise difficult to access. Marine microalgae such *as P. tricornutum* actively regulate their ionic and chemical microenvironment through membrane transport, metabolism and secretion of extracellular material. In the present system, these processes do not need to produce large deterministic currents to become measurable. Instead, they can be detected through their stochastic modulation of interfacial admittance and distributed transport pathways within the porous mixed conductor. Importantly, the lower stochastic activity observed for EASW‐only control electrodes demonstrates that nonspecific contributions from the electrolyte or electrode alone cannot account for the measured electrical signatures, indicating that the stochastic fluctuations originate predominantly from interactions between adhered *P. tricornutum* and the porous PU/PEDOT:PSS interface. This establishes non‐equilibrium noise analysis as a complementary bioelectronic observable for microbial ecophysiology. Having demonstrated that the measured fluctuations predominantly originate from the living biohybrid interface, an important next question is how these extracellular electrical signatures relate to intracellular physiological activity. To address this challenge, future studies combining extracellular noise analysis with intracellular optical reporters will be particularly valuable. Although the present work demonstrates robust population‐level bioelectrical signatures and establishes that the applied electrochemical conditions preserve high cellular viability, directly correlating intracellular signalling events with extracellular stochastic electrical activity represents an important next step toward resolving the biological origins of these fluctuations. Genetically encoded fluorescent calcium biosensors have enabled real‐time visualization of rapid signalling responses in living diatoms with single‐cell resolution, revealing stimulus‐specific intracellular Ca^2+^ dynamics that cannot readily be captured using conventional viability stains alone [12, 43]. Integrating such optical reporters with the organic bioelectronic platform developed here would enable simultaneous monitoring of intracellular signalling, cellular physiological state and extracellular electrical fluctuations, providing a powerful multimodal approach for elucidating the mechanisms underlying microbial bioelectrical activity.

Beyond correlating intracellular signalling with extracellular fluctuations, an equally important direction is to investigate how environmental perturbations shape these stochastic electrical dynamics. The present study was intentionally performed under a single, well‐controlled physiological condition using axenic cultures to isolate the contribution of microbial activity to the measured stochastic electrical fluctuations. We recognise that photosynthetic organisms exhibit pronounced light‐dependent physiological and electrochemical responses and therefore the influence of illumination, circadian regulation and transitions between light and dark conditions on stochastic electrical noise represents an important direction for future investigation. Although these effects fall beyond the scope of the present work, extending the present methodology to dynamically varying environmental conditions could provide further insight into the coupling between microbial physiology and stochastic bioelectrochemical fluctuations. Additionally, while the present work compares electrodes following identical incubation periods, future studies involving periodic impedance spectroscopy and noise measurements throughout culture development will enable quantitative assessment of electrode ageing, biofouling and temporal evolution of the bioelectronic interface.

Finally, interpretation of these stochastic electrical fluctuations requires confidence that the electrochemical protocol itself does not induce widespread cellular damage. Additional membrane integrity experiments therefore provide an important control. Confocal fluorescence imaging using SYTOX^TM^ Green following prolonged electrical stimulation revealed no evidence of widespread membrane permeabilization, even after continuous exposure to 500 mV for 4 h (Figure S10). Although these measurements do not directly assess metabolic activity, they demonstrate that the electrical stimulation protocol does not produce extensive membrane damage under the conditions employed in this work. Consequently, the observed stochastic electrical fluctuations are unlikely to arise simply from cell lysis but instead reflect dynamic electrochemical interactions occurring at viable biohybrid interfaces.

## 3 Conclusions

We developed mesoporous PU/PEDOT:PSS electrodes that form volumetrically coupled, ultra‐low‐impedance mixed ionic‐electronic interfaces capable of resolving stochastic electrochemical fluctuations in aqueous environments. Electrochemical impedance spectroscopy reveals that these systems are governed by distributed interfacial dynamics associated with ionic transport within the porous conducting network. Under DC polarization, these dynamics give rise to broadband 1/f current noise that significantly exceeds the equilibrium thermal noise predicted from impedance measurements, demonstrating operation in a non‐equilibrium regime.

In the presence of axenic cultures of *Phaeodactylum tricornutum*, both the amplitude of current fluctuations and the occurrence rate of transient stochastic events increase relative to electrolyte‐only controls, demonstrating that living diatoms actively modulate the electrochemical dynamics of the interface. Complementary microscopy, cyclic voltammetry, transmission‐line modelling and pharmacological inhibition with tetraethylammonium (TEA) further show that this enhanced stochastic activity is associated with physiologically active, membrane‐dependent processes while reorganizing distributed ionic transport and charging pathways within the porous scaffold without disrupting its electrochemical functionality. These findings establish porous mixed ionic‐electronic conductors as volumetric transducers that convert distributed ionic and electrochemical dynamics within microbial environments into measurable stochastic electrical signals. More broadly, this work represents an important step toward probing population‐level stochastic bioelectrochemical dynamics in marine systems, opening new opportunities for microbial ecophysiology, environmental sensing, biogeochemical modelling, high‐throughput functional screening and future investigations of electrical interactions within complex microbial communities.

## 4 Methods

### 4.1 Fabrication of Porous PU/PEDOT:PSS Electrodes

Commercial polyurethane (PU) sponge (Eurospuma, 3049PR) was laser‐cut into cylindrical samples (diameter 7.7 mm, height 7.0 mm) and semi‐circular samples (diameter 10.0 mm, height 12.0 mm, thickness 4.0 mm), used as porous scaffolds for the working and counter electrodes, respectively. The PU structures were first washed with soap and distilled water, followed by sonication for 10 min in a 1:1 (v/v) mixture of acetone and distilled water to remove residual contaminants. The samples were then dried with compressed air. Immediately before coating, the PU structures were treated in an oxygen plasma chamber (Diener Atto, 40 kHz, 80 W) for 60 s to further clean the surface and increase hydrophilicity.

The PEDOT:PSS coating solution used for the first and second dip‐coating cycles was prepared by mixing 190 mL of PEDOT:PSS (Clevios PH1000, 94.25 vol.%, Heraeus Precious Metals GmbH & Co., Germany), 10 mL of ethylene glycol (Supelco, USA), 0.4 mL of poly(ethylene glycol) diglycidyl ether (PEGDGE, Sigma–Aldrich, USA) and 90 µL of Triton X‐100 (Thermo Scientific Chemicals, USA). The mixture was stirred at 200 rpm for 6 h to ensure homogenization. For the first and second coating cycles, the PU structures were immersed in this PEDOT:PSS mixture and agitated overnight on an orbital shaker at 200 rpm. The beaker containing the infiltrated PU cylinders was then placed in a vacuum oven (Thermo Scientific VT6025). A vacuum of 50 mbar was applied for 5 min and followed by a slow return to atmospheric pressure. This vacuum‐release cycle was repeated two additional times to remove trapped air and improve infiltration within the porous network.

After infiltration, the PU structures were transferred to a cylindrical grill and thermally treated in a muffle furnace (Thermo Scientific F30428C‐80) at 60°C for 10 min, 90°C for 10 min and 130°C for 30 min while being continuously rotated at 5 rpm. The samples were then allowed to cool to room temperature in a fume hood. This infiltration and thermal‐curing sequence was repeated to obtain the second coating. For the third and final coating cycle, a second PEDOT:PSS formulation was used as a post‐treatment coating. This solution was prepared by mixing 127.7 mL of PEDOT:PSS, 15 mL of dimethyl sulfoxide (DMSO, Carlo Erba, Italy) and 7.5 mL of ethylene glycol, followed by stirring at 200 rpm for 6 h. The twice‐coated PU structures were immersed in this post‐treatment solution for 5 s and then subjected to the same thermal‐treatment sequence (60°C for 10 min, 90°C for 10 min and 130°C for 30 min, with continuous rotation at 5 rpm). After cooling, the coated structures were immersed in 1 M sulfuric acid (Honeywell‐Fluka, Germany) for 45 s under gentle agitation, rinsed thoroughly with distilled water, immersed in 5 m sulfuric acid for 10 s, rinsed again thoroughly with distilled water and finally thermally treated at 130°C for 30 min. To evaluate the reproducibility of the acid‐treatment protocol, three independently fabricated porous PU/PEDOT:PSS electrodes were prepared for each treatment condition and characterized using current‐voltage measurements and electrochemical impedance spectroscopy (Figure S4). Mild acid treatment increased the electrical conductivity while maintaining reproducible impedance characteristics across independently fabricated electrodes. Among the conditions investigated, treatment with 5 m H_2_SO_4_ for 10 s provided the greatest improvement in conductivity while preserving low impedance, whereas prolonged exposure (5 m H_2_SO_4_ for 5 min) increased impedance and was accompanied by localized disruption of the PEDOT:PSS coating observed by SEM, indicating the onset of over‐treatment. Unless otherwise stated, the electrodes used throughout the study correspond to this acid‐treated three‐coating configuration. Electrode mass, median pore diameter and porosity were quantified before and after coatings to assess structural preservation of the porous scaffold using mercury porosimetry (Micromeritics AutoPore IV 9500).

Planar PEDOT:PSS electrodes were also fabricated for benchmarking on lithographically patterned glass substrates to provide a planar control with similar conducting polymer chemistry as the mesoporous PU/PEDOT:PSS electrodes. Borosilicate glass wafers (2 mm thickness, 100 mm diameter; Präzisions Glas & Optik GmbH, Germany) were patterned with eight circular Au working electrodes (0.204 mm^2^) and corresponding semi‐ring Au counter electrodes (6 mm^2^) separated by approximately 1.9 mm. Metal layers consisted of a 10 nm Ti adhesion layer followed by 50 nm Au deposited through a shadow mask. An aqueous electropolymerization solution containing EDOT (0.1% w/v; Sigma–Aldrich) and sodium polystyrene sulfonate (PSSNa, 0.7% w/v; Sigma–Aldrich) was prepared in deionised water, stirred for 3 h at 250 rpm and allowed to equilibrate for 1 h before use. PEDOT:PSS films were deposited potentiostatically at 0.85 V vs. Ag/AgCl for 180 s using a three‐electrode configuration consisting of the planar Au working electrode, a Pt counter electrode and an Ag/AgCl reference electrode (Autolab PGSTAT302N, Metrohm) [44]. Then, axenic *P. tricornutum* cultures were diluted in ESAW medium to 1 × 10^7^ cells mL^−1^. Each electrode well was filled with 200 µL of cell suspension, sealed with a transparent lid and incubated under identical culture conditions for two weeks prior to electrochemical measurements.

### 4.2 Electrode Assembly and Electrical Contacts

After acid treatment, the PEDOT:PSS‐coated cylindrical structures, designated as working electrodes, were fixed onto thermally evaporated circular Au pads (area 38.5 mm^2^) using 20 µL of silver conductive epoxy (EP77M‐FMed, Master Bond, USA). PEDOT:PSS‐coated semi‐circular structures, designated as counter electrodes, were fixed onto semi‐ring‐shaped Au pads (area 90.6 mm^2^) using 20 µL of the same silver conductive epoxy. The assembled structures were left to dry at room temperature for 12 h inside a sterile flow chamber (Herasafe 2030i, Thermo Fisher Scientific, USA). The metal contact layers were deposited through a shadow mask using an Edwards High Vacuum 4P thermal evaporator and consisted of a 10 nm Ti adhesion layer followed by a 50 nm Au layer on 2 mm thick borosilicate glass slides (Präzisions Glas & Optik GmbH, Germany), as described previously [1, 2].

### 4.3 Estimation of Geometric Parameters and Electroactive Surface Area

The volume of PEDOT:PSS within the porous scaffold was estimated from the measured electrode mass assuming a PEDOT:PSS density of 1 g cm^−3^. The solid volume was calculated as:

(4) $$V_{\mathrm{solid}}=\frac{m}{\rho }\;$$

where *m* is the measured mass and ρ the density of PEDOT:PSS.

The external geometric surface area of the cylindrical electrodes was approximated as:

(5) $$A_{\mathrm{external}}=2\pi rh+2\pi r^{2}$$

where *r* and *h* correspond to the measured radius and height of the electrode.

The internal surface area of the porous PEDOT:PSS coating was estimated using the Brunauer‐Emmett‐Teller (BET) specific surface area of PEDOT:PSS foams (2.83 m^2^ g^−1^). The internal area was calculated as:

(6) $$A_{\mathrm{internal}}=BET\cdot m$$

The total electroactive surface area was therefore calculated as:

(7) $$A_{\mathrm{total}}=A_{\mathrm{external}}+A_{\mathrm{internal}}$$

The total capacitance was estimated using the volumetric capacitance of PEDOT:PSS foams (21 F cm^−3^):

(8) $$C_{\mathrm{total}}=C_{\mathrm{vol}}\;\cdot V_{\mathrm{solid}}$$

and the areal capacitance was calculated as:

(9) $$C_{\mathrm{areal}}=\frac{C_{\mathrm{total}}}{A_{\mathrm{total}}}\;$$

### 4.4 Electrical Characterization

The intrinsic conductivity of PEDOT:PSS films was measured on spin‐coated thin films (165 nm thickness) using the four‐point probe van der Pauw method, yielding a conductivity of approximately 1600 S cm^−1^. Porous PU/PEDOT:PSS cylinders were electrically characterized using a Keysight B1500 parameter analyser in a two‐terminal configuration. The flat circular faces of each cylinder were contacted using silver paste bonded to copper pads on a printed circuit board to ensure full‐area electrical contact. Current was driven along the cylinder axis. Current‐voltage measurements were performed by sweeping the applied voltage from 0 to 75 mV in 10 mV increments. For each electrode configuration, three independent I‐V measurements were recorded.

Conductance was extracted from linear least‐squares fits using:

(10) I=G·V+b

where *G* is the conductance and *b* accounts for instrumental offsets. Resistance was calculated as *R*  =  1/*G* and the effective conductivity was determined from:

(11) $$\sigma =\frac{L}{R\cdot A}\;$$

where *L* is the electrode height and *A*the cross‐sectional area.

### 4.5 Electrochemical and Chronoamperometric Measurements

Electrochemical measurements were performed in artificial seawater (EASW) electrolyte and in axenic cultures of *P. tricornutum* following 11 days of incubation. The projected circular area was 0.376 cm^2^ and was used for normalization of current density. Electrochemical impedance spectroscopy (EIS) and cyclic voltammetry (CV) were performed using a PalmSens EmStat4S potentiostat in a three‐electrode configuration. The porous PU/PEDOT:PSS electrode served as the working electrode, an Ag/AgCl electrode as the reference electrode and a platinum mesh (15×15 mm) as the counter electrode. Electrochemical impedance spectroscopy was performed using a 10 mV AC small signal over the frequency range 10^−4^ − 10^2^ Hz. Impedance spectra were fitted using an equivalent circuit consisting of a series resistance in series with a parallel combination of a charge‐transfer resistance and a constant phase element as in Equation 1. Effective double‐layer capacitance values were extracted from the CPE parameters using the Hsu–Mansfeld [45] approximation:

(12) $$C_{dl}=Q^{1/n}\;\cdot R_{ct}^{\left(1-n\right)/n}$$

Electrical fluctuations at the electrode‐electrolyte interface were measured using chronoamperometry under controlled DC polarization. A porous PU/PEDOT:PSS working electrode was biased with a DC potential (0–500 mV) relative to the counter electrode and connected to a low‐noise transimpedance amplifier SRS570 to record current fluctuations. Current traces were acquired continuously and analysed using MATLAB. Only steady‐state portions of the recordings were used for spectral analysis, with the initial capacitive relaxation following bias application excluded.

Current fluctuations were analysed in the time and frequency domain. Raw current signals were first detrended by subtracting a moving median baseline with window = 15 s, to remove slow drift. The resulting signal was then smoothed using a moving average filter with a 0.2 s window to suppress high‐frequency noise while preserving transient features. For spectra analysis, the current signal was divided into overlapping segments with 50% overlap, windowed using a Hann window and Fourier transformed. The resulting spectra were averaged to obtain the final PSD for each measurement condition, on at least two distinct samples. The spectra were analysed over the frequency range 1–50 Hz to avoid low‐frequency drift and high‐frequency instrumental noise. Spectra were fitted using the power‐law in Equation 13:

(13) $$S_{I}\left(f\right)=Af^{-\alpha }$$

where *A* is the fluctuation amplitude and α the spectral exponent.

The experimentally measured spectra were compared with the equilibrium thermal noise predicted from the fluctuation‐dissipation relation (Figure 3C) given by Equation 3. Power spectral densities (PSDs) were calculated over the frequency range of 10^−3^–50 Hz using Welch's method. For quantitative comparison between experimental conditions, the power‐law exponent (α) was determined by fitting the PSD to *S_I_* (*f*)∝*f* ^−α^ over the 1–50 Hz frequency interval. This fitting range was selected to provide a robust estimate of the 1/*f* ^α^ behavior while minimizing edge effects associated with the finite duration of the recordings at the lowest measurable frequencies.

Transient current events were identified from the current‐time recordings following baseline correction using a 30 s moving median filter. To establish an objective detection criterion independent of the biological recordings, the baseline noise level was estimated from the corresponding media‐only control acquired under identical experimental conditions. The noise amplitude was calculated using the median absolute deviation (MAD), which was converted to an equivalent standard deviation according to σ  =  1.4826 × *MAD*. A conservative detection threshold was then defined as six times the estimated baseline noise (6σ). Positive and negative current transients were detected independently using MATLAB's findpeaks function with a minimum peak prominence equal to the detection threshold, a minimum peak separation of 0.25 s, and a minimum peak width of 10 ms. Consecutive peaks satisfying these criteria were considered individual stochastic electrical events. Event rate was calculated as the total number of detected events per minute, while inter‐spike intervals were obtained from the temporal separation between successive detected events. For graphical representation, inter‐spike intervals exceeding 60 s were clipped to 60 s to avoid distortion by rare long‐duration intervals.

For comparison between mesoporous PU/PEDOT:PSS and planar PEDOT:PSS electrodes, identical event‐detection parameters and signal‐processing procedures were applied to all recordings. To account for intrinsic differences in electrode background activity, recordings obtained from *P. tricornutum* cultures were normalised to architecture‐specific medium controls acquired under identical experimental conditions. The primary metrics used for comparison were the excess event rate (cell—medium), median event prominence and the ratio of integrated low‐frequency PSD (0.1–10 Hz) between cellular and corresponding medium recordings. These architecture‐normalised metrics isolate biologically generated stochastic electrical activity from the intrinsic electrical characteristics of each electrode and were used for the quantitative comparison presented in Figure S7.

### 4.6 Cyclic Voltammetry

Cyclic voltammetry (CV) measurements were conducted over a potential window of ‐0.5–0.5 V at scan rates of 1, 5, 10, 25, 50 and 100 mV s^−1^. For each scan rate, four consecutive cycles were recorded. To minimize the influence of initial conditioning effects, including electrode wetting and transient stabilization of ionic pathways within the porous scaffold, the first scan was excluded from quantitative analysis. Mean CV traces and standard deviations were calculated from scans 2–4 using a normalized path‐length interpolation approach to account for variations in sampling density. Peak‐current scaling was evaluated by extracting the maximum absolute current density from each scan and fitting the relation *i*  =  *a* · *v^b^* in log‐log space [15, 46]. The exponent b was used to assess the dominant charge‐storage regime. Fixed‐potential analysis was performed by interpolating current density at selected potentials (0.00, 0.20 and 0.40 V) separately for forward and reverse branches, enabling assessment of potential‐dependent scaling behavior.

### 4.7 Transmission‐Line Modelling

Impedance spectra were acquired over the frequency range 10^−3^–10^5^ Hz under open‐circuit conditions. A transmission‐line description was employed to capture distributed ionic‐electronic transport within the porous electrode, which cannot be represented by a single lumped interface. The complex impedance *Z*(ω) was analysed using a finite‐length transmission‐line model describing distributed ionic‐electronic transport in porous mixed conductors. The electrode was modelled as a series resistance (*R_s_* + *R_c_*) connected to a transmission line with distributed resistance *r_t_* and pore admittance *Y_p_* (ω) defined following established transmission‐line circuits for porous conductors [47, 48]:

(14) $$Y_{p}\;\left(\omega \right)=Q_{p}\;*{\left(j*\omega \right)}^{n_{p}}+\frac{1}{R_{leak}}$$

The distal end of the transmission line was terminated by a parallel combination of a resistance Rt and a constant‐phase element:

(15) $$Y_{t}\;\left(\omega \right)=\frac{1}{R_{t}}+Q_{t}*{\left(j*\omega \right)}^{n_{t}}$$

The total impedance of the transmission line is given by:

(16) $$Z\;\left(\omega \right)=R_{s}+R_{c}+Z_{TLM}\left(\omega \right)$$

where,

(17) $$Z_{0\left(\omega \right)}=sqrt\left(\frac{r_{t}}{Y_{p}\left(\omega \right)}\right)$$

(18) $$\gamma \;\left(\omega \right)=sqrt\left(r_{t}*Y_{p}\left(\omega \right)\right)$$

(19) $$Z_{term}\;\left(\omega \right)=\frac{1}{Y_{t}\left(\omega \right)}\;$$

(20) $$Z_{TLM}\;\left(\omega \right)=Z_{0}\;\left(\omega \right)*\frac{Z_{term}\left(\omega \right)+Z_{0}\left(\omega \right)*\tanh\left(\gamma \left(\omega \right)\right)}{Z_{0}\left(\omega \right)+Z_{term}\left(\omega \right)*\tanh\left(\gamma \left(\omega \right)\right)}$$

Here, *j* = √(−1) and ω = 2π*f*. *Q*_p_ and *Q*_t_ are constant‐phase element parameters with exponents *n_p_* and *n_t_*, respectively. The variable *r_t_* represents the distributed ionic resistance along electrolyte‐filled pores, while *Y_p_* describes the local interfacial admittance associated with ionic‐electronic coupling within the porous PEDOT:PSS network.

### 4.8 Biological Measurements

Pt15 cultures were made axenic using a cocktail of antibiotics (ampicillin 100 µg/mL, chloramphenicol 15 µg/mL and streptomycin 100 µg/mL). Axenicity was verified by a growth test on a peptone‐rich medium, light microscopy with DAPI staining, and 16S PCR amplification of DNA extracted from Pt15 for bacterial detection. All tests confirmed that the Pt15 cells were axenic. The 16S rRNA primers used were: fD1 AGAGTTTGATCCTGGCTCAG and rD1 AAGGAGGTGATCCAGCC.

Axenic cultures of the marine diatom Pt15 were seeded into the electrochemical chamber at a density of 10^7^ cells mL^−1^ and allowed to adhere to the porous PU/PEDOT:PSS electrodes for at least 11 days prior to measurement. During incubation, cultures were maintained in EASW medium in an ARALAB growth chamber at 18°C under a 12 h light / 12 h dark cycle with 30 µmol/m^2^/s provided by cool white daylight fluorescent lamps. Before each electrochemical measurement, the incubated Pt15‐electrode assemblies were carefully removed from the growth chamber and transferred to a Faraday cage for low‐noise electrical measurements. Unless otherwise stated, all recordings were performed in the dark. Axenicity was assessed after experiments by scanning electron microscopy as shown in Figure 5B,C. Electrical measurements were performed on both Pt15‐electrode systems and EASW‐only controls under otherwise identical conditions to isolate biological contributions to the measured stochastic fluctuations.

To monitor culture growth, Pt15 cell density was periodically determined using Mallassez chamber counting under a light microscope throughout cultivation in artificial seawater supplemented with 20 µM nitrate, under an irradiance of 50 µmol *photons* m^−2^ s^−1^ and a 12/12 h light dark photoperiod. The resulting growth curve showed the characteristic growth pattern of batch cultures [31, 39, 49, 50] and is provided in Figure S8.

During pharmacological inhibition experiments, a 250 mm stock solution of tetraethylammonium chloride (TEA; Sigma–Aldrich, Germany) was prepared in deionized water. A volume of 120 µL was added directly to each well containing 3 mL culture medium and the cell‐seeded porous working electrode, yielding a final TEA concentration of 10 mM. Electrochemical recordings were continued following TEA addition, and paired analyses were performed using recording windows acquired approximately 15–20 min after treatment.

### 4.9 Confocal Assessment of Membrane Integrity Following Electrical Stimulation

To evaluate whether prolonged electrical stimulation affected membrane integrity, axenic *P. tricornutum* cultures (10^7^ cells mL^−1^) colonizing the porous PU/PEDOT:PSS scaffold were exposed to constant DC biases of 0, 100, 300 or 500 mV for 4 h, corresponding to the stimulation conditions employed throughout the stochastic electrical measurements. Following stimulation, samples were incubated for 5 min with 0.5 µm SYTOX^TM^ Green nucleic acid stain (Thermo Fisher Scientific) prepared in Milli‐Q water. Confocal fluorescence imaging was performed using a Carl Zeiss LSM 710 NLO laser‐scanning microscope equipped with a W Plan‐Apochromat 20×/1.0 objective. SYTOX^TM^ Green fluorescence was excited using the 488 nm argon laser line and emission collected between 494 and 563 nm. Chlorophyll autofluorescence was excited using a 561 nm diode laser and collected between 567 and 656 nm. Three independent fields of view were acquired for each stimulation condition.

## Author Contributions

D.M.S.S. investigation, data curation, formal analysis, visualization, Writing – original draft. L.C., F.L.B., R.A., L.‐J.L.‐A. methodology, validation. K.A., L.T. methodology, validation, writing (review and editing). P.R. conceptualization, methodology, supervision, funding acquisition, writing (review and editing).

## Conflicts of Interest

The authors declare no conflicts of interest.

## Supporting information




**Supporting File**: advs77341‐sup‐0001‐SuppMat.docx.

## Data Availability

The data that support the findings of this study are available from the corresponding author upon reasonable request.
