# Supplementary material for: Mesoporous PU/PEDOT:PSS Electrodes Reveal Population‐Level Stochastic Bioelectrical Dynamics in Marine Diatoms
Source: Adv Sci (Weinh). 2026 Sep 5:e77341. Online ahead of print. doi: 10.1002/advs.77341 (PMC13546024; doi:10.1002/advs.77341)
Supplement: Supplementary file 1 [file ADVS-9999-e77341-s001.docx]

**Supplementary**

Figure S1 - Electrode mounting and electrical contacting procedure for I-V traces. Copper plates were sectioned into square substrates of 11x11 mm. Each substrate was mechanically polished using 2000-grit silicon carbide paper to remove surface oxides and irregularities, thereby ensuring low interfacial contact resistance. Residual particulates were eliminated by air drying, followed by rinsing with isopropyl alcohol. A tinned copper wire was soldered to one corner of each substrate to provide a stable and reproducible electrical connection. Subsequently, 200 µL of silver conductive paint (Electrolube SCP50G) was dispensed onto the prepared copper surface. The porous PEDOT:PSS electrode was carefully positioned on top of the silver layer, ensuring uniform contact across the interface. The same contacting procedure was repeated on the opposite side of the electrode to ensure symmetric electrical interfacing.

**Figure S2 - Structural characterization of porous PU/PEDOT:PSS electrodes during dip-coating and acid post-treatment.** (A) Electrode mass as a function of processing step, showing progressive PEDOT:PSS accumulation from 1x to 3x coating cycles and negligible change following sulfuric-acid post-treatment. (B) Median pore diameter, showing a reduction relative to the uncoated polyurethane scaffold consistent with conformal coating of pore walls by PEDOT:PSS, while maintaining an open interconnected pore network. (C) Porosity of the electrodes during processing. Although pore diameter decreases with increasing coating cycles, the overall porosity remains high. The acid-treated electrodes remains comparable to the 3x condition within experimental variation, indicating integrity preservation. Data are shown as mean ± standard deviation (n = 5).

**Figure S3 - Electrochemical impedance spectroscopy of porous PU/PEDOT:PSS electrodes as a function of dip-coating cycles.** Electrochemical impedance spectra of porous PU/PEDOT:PSS electrodes prepared using one to five PEDOT:PSS dip-coating cycles.
(A) The impedance magnitude $\mid Z\mid$as a function of frequency for electrodes coated one (1x) to five (5x) times. Symbols denote experimental data and solid lines correspond to fits using the Randles-type equivalent circuit. Increasing the number of dip-coating cycles from one to three leads to a progressive reduction in impedance across the measured frequency range, reflecting improved electronic connectivity and enhanced interfacial charge-transfer kinetics. Additional coating cycles (four and five) reverse this trend, consistent with excessive film thickness, increased tortuosity, partial pore clogging and reduced bulk transport. (B) Corresponding phase spectra with model fits for the same electrodes. The inset shows the Nyquist representation (-Z’’ Vs Z’), highlighting the decrease in semicircle diameter ($R_{ct}$) from one to three dip-coating cycles, followed by an increase for thicker coatings (four and five cycles). Together, these data identify three dip-coating cycles as the optimal condition for minimizing impedance and maximizing charge-transfer efficiency while maintaining low series resistance.

**Figure S4. Effect of acid treatment on the morphology and electrical reproducibility of porous PU/PEDOT:PSS electrodes**. (A-D) Representative scanning electron microscopy (SEM) images of untreated electrodes and electrodes treated with 1 M H_2_SO_4_ for 45 s, 5 M H_2_SO_4_ for 10 s, and 5 M H_2_SO_4_ for 5 min, respectively. Mild acid treatments preserve the overall macroporous architecture of the polyurethane scaffold and the continuity of the conductive coating. In contrast, prolonged treatment with 5 M H_2_SO_4_ for 5 min produces localized surface roughening and partial disruption of the PEDOT:PSS coating, indicating the onset of over-treatment. (E) Current-voltage characteristics (mean ± SD, n = 3 independent electrodes) demonstrating the increase in electrical conductivity following acid treatment, with the greatest improvement obtained after treatment with 5 M H_2_SO_4_ for 10 s. (F) Impedance magnitude (|Z|) as a function of frequency for the four treatment conditions, showing the reproducibility of the electrical response across independently fabricated electrodes. Symbols represent the mean and error bars denote the standard deviation (n = 3). (G) Median impedance magnitude calculated over the measured frequency range (0.01 Hz-200 kHz) for each independent electrode. Open circles indicate the group mean and error bars represent the standard deviation.

Figure S5 - Bias-dependent electrochemical impedance spectroscopy of porous PEDOT:PSS electrodes submerged in EASW medium. Measured impedance spectra (black circles) and equivalent circuit fits (red lines) obtained at applied DC biases ranging from 0 to 500 mV in electrolyte solution, in each different line. For each bias condition three representations are shown over the three columns, *e.g.,* the left column shows the Bode magnitude plots exhibiting the absolute impedance $\boldsymbol{\mid}\boldsymbol{Z}\boldsymbol{\mid}$ as a function of frequency, whereas the middle column shows the negative phase angle ($\boldsymbol{-}\boldsymbol{\theta)}$ as a function of frequency and finally, the right column shows the Nyquist representation of the impedance ($\boldsymbol{-}\boldsymbol{Z}^{\mathbf{''}}$ Vs $\boldsymbol{Z}^{\mathbf{'}}$). The spectra were fitted using a Randles-type equivalent circuit Rs + (Rct || CPE). Extracted parameters are summarized in Table S3.

**Figure S6 - Temperature-dependent impedance reveals distributed relaxation processes in porous PEDOT:PSS electrodes.** (A) Impedance modulus ∣Z∣ of the porous PEDOT:PSS electrode measured in 100 mM KCl electrolyte between 10 and 40 °C. A reproducible deviation from the dominant constant-phase-element behaviour appears in the low-frequency region (shaded area between 0.1-1 Hz), visible as a broad shoulder in ∣Z∣. This feature indicates the presence of an additional slow relaxation process superimposed on the main charge-transfer/CPE response. The inset shows the corresponding phase spectra θ over the frequency range 10^-3^-10^4^ Hz. The vertical dotted line marks the reference frequency $f_{0}=0.4$ Hz used for fixed-frequency comparison of temperature-dependent loss characteristics. (B) Loss tangent $\tan(\alpha)=(-Z^{''})/Z^{'}$ plotted over the low-frequency region. In contrast to a simple RC relaxation, which would produce a sharp peak in tan(α), the response remains broad and monotonic, indicating that the underlying process is distributed over a wide range of time constants. The inset shows tan(α) evaluated at $f_{0}=0.4$ Hz as a function of temperature, revealing systematic temperature dependence of the low-frequency polarization losses. This behaviour is consistent with thermally activated ionic redistribution and interfacial polarization within the porous PEDOT:PSS network.

**Table S1 - Equivalent-circuit parameters extracted from electrochemical impedance spectroscopy of PU/PEDOT:PSS electrodes as a function of dip-coating cycles.** Equivalent-circuit parameters obtained by fitting electrochemical impedance spectroscopy (EIS) data of porous PU/PEDOT:PSS electrodes prepared with one to five PEDOT:PSS dip-coating cycles. Values are reported as mean ± standard deviation (n = 3). Both $R_{s}$and $R_{ct}$ exhibit a clear minimum at three dip-coating cycles, indicating optimal electronic percolation and interfacial charge-transfer kinetics. Additional coating cycles (four and five) lead to increased resistive components, consistent with excessive film thickness, increased tortuosity and reduced vertical transport within the PEDOT:PSS network. $C_{\mathrm{dl}}$ estimates for 4x and 5x should be interpreted cautiously due to lumped-element limitations in highly tortuous structures.

| **Dip-coating cycle** | **R_s_ (Ω)** | **R_ct_ (kΩ)** | **Q (F·s⁽ⁿ-¹⁾)** | **n** | **C_dl_ (mF)** |
| --- | --- | --- | --- | --- | --- |
| 1x | 309.2 ± 34.1 | 129.6 ± 15.6 | 0.05351 ± 0.00642 | 0.970 ± 0.012 | 70.34 ± 11.78 |
| 2x | 125.5 ± 15.1 | 39.34 ± 6.72 | 0.10000 ± 0.01200 | 0.978 ± 0.012 | 120.47 ± 19.37 |
| 3x | 71.77 ± 8.31 | 16.90 ± 3.03 | 0.17800 ± 0.02136 | 0.969 ± 0.012 | 229.98 ± 36.97 |
| 4x | 96.0 ± 11.5 | 20.77 ± 0.25 | 0.203 ± 0.024 | 0.965 ± 0.012 | 274.77 ± 44.80 |
| 5x | 116.1 ± 13.9 | 28.42 ± 0.34 | 0.262 ± 0.031 | 0.972 ± 0.012 | 338.72 ± 56.31 |

**Supplementary Figure S7** - **Comparison of mesoporous PU/PEDOT:PSS and planar PEDOT:PSS electrodes under identical biological conditions.** Biological activity was quantified after normalisation to paired architecture-specific media controls recorded under identical experimental conditions. This normalisation removes the architecture-dependent electrical background, allowing direct comparison of the biological contribution measured by each electrode type. (A) Excess event rate, calculated as the difference between cellular and media event frequencies. (B) Median transient-event prominence. (C) Integrated power spectral density (PSD) ratio between cellular recordings and paired media controls over 0.1-10 Hz. Individual electrodes are shown as coloured symbols, while open circles indicate group means and error bars denote ±1 standard deviation. Across all three complementary metrics, 3D porous PU/PEDOT:PSS electrodes exhibit larger biological responses than planar PEDOT:PSS electrodes after accounting for architecture-specific background signals.

**Figure S8 -** **Growth characteristics of the axenic *Phaeodactylum tricornutum* Pt15 culture used throughout this study**. Cell density was monitored by haemocytometer counting during batch cultivation. Grey circles represent individual haemocytometer counts, while open circles indicate the mean ± standard deviation at each sampling time. The solid black line corresponds to the best-fit logistic growth model, and the shaded pink region represents the 95% bootstrap confidence band of the fitted curve. The logistic model was used as a descriptive model of batch population growth approaching a finite carrying capacity and was not used for subsequent statistical analysis. The growth profile exhibits the expected transition from exponential growth to stationary phase, reaching a stable population of approximately 2x10^6^ cells mL^-1^ after approximately 10-14 days.

**Figure S9 - Scanning electron microscopy (SEM) images of porous PU/PEDOT:PSS electrodes after incubation with *Phaeodactylum tricornutum* suspensions at increasing cell densities**. (A) 10^5^ cells mL^-1^, (B) 10^7^ cells mL^-1^ and (C) 10^8^ cells mL^-1^. Images show progressively greater occupation of the porous electrode surface with increasing cell density. At the highest density, the scaffold appears extensively covered by adhered biomass, consistent with surface saturation of the electrode. Scale bars: 50 μm.

**Figure S10 - Confocal fluorescence assessment of diatom membrane integrity following prolonged electrical stimulation.** Representative confocal laser-scanning microscopy images of axenic *Phaeodactylum tricornutum* (10^7^ cells mL^-1^) colonising the porous PU/PEDOT:PSS scaffold after 4 h under the different electrical stimulation conditions used in this study. Rows correspond to (A-C) no electrical stimulation (0 mV), (D-F) 100 mV, (G-I) 300 mV, (J-L) 500 mV (field 1) and (M-O) 500 mV (field 2). For each condition, three independent fields of view were acquired from the same biohybrid electrode. The first column (A, D, G, J, M) shows SYTOX^TM^ Green fluorescence, identifying cells with compromised membrane integrity. The second column (B, E, H, K, N) shows chlorophyll autofluorescence, visualising the total diatom population. The third column (C, F, I, L, O) shows the merged images. White arrows indicate representative SYTOX-positive cells. Across all stimulation conditions, only occasional SYTOX-positive cells were observed, with no qualitative increase in membrane-compromised cells following continuous electrical stimulation up to 500 mV for 4 h. These observations indicate that the electrical stimulation protocol employed throughout this study preserves the overall integrity of the diatom population. Scale bars = 20 µm.

**Figure S11 - Transmission-line analysis of impedance reveals biological modification of distributed transport pathways.** (A) Impedance magnitude (|Z|) of porous electrodes measured in artificial seawater (EASW) in blue and in axenic *P. tricornutum* cultures in red, together with fits to a terminated finite-length transmission-line model. (B) Negative phase angle as a function of frequency, highlighting distinct relaxation processes in each condition. Inset: Nyquist representation of the same data, emphasizing differences in low-frequency impedance trajectories. The model consists of a series resistance ($R_{s}+R_{C}$) coupled to a finite-length transmission line describing distributed ionic-electronic transport within the porous PEDOT:PSS network, with pore admittance represented by a constant-phase element (CPE) and an optional leakage pathway. The distal end of the transmission line is terminated by a parallel combination of a resistance ($R_{t}$) and a CPE ($Q_{t},n_{t}$), capturing slow relaxation processes associated with deep pore regions. Fits reveal that biological colonization primarily modifies distributed pore admittance and terminal relaxation behaviour, while no significant leakage pathway is required.

**Table S2 - Transmission-line model parameters for porous PEDOT:PSS electrodes**

| **Parameter** | **Unit** | **Media (EASW)** | **Cells (Pt15 axenic)** |
| --- | --- | --- | --- |
| $R_{s}$ | Ω | 15.0 | 19.5 |
| $R_{c}$ | Ω | 10^-3^ | 10^-3^ |
| $r_{t}$ | Ω | 10.3 | 95.0 |
| $Q_{p}$ | S·sⁿ | 1.07×10^-5^ | 9.71×10^-5^ |
| $n_{p}$ | - | 0.83 | 0.85 |
| $R_{leak}$ | Ω | >10^10^ | >10^10^ |
| $Q_{t}$ | S·sⁿ | 0.157 | 0.233 |
| $n_{t}$ | - | 0.92 | 1.00 |
| $R_{t}$ | Ω | 2059 | 748 |

**Table S3** - **Equivalent circuit parameters obtained from fitting the impedance spectra at different applied biases using the model** $\boldsymbol{R}_{\boldsymbol{s}}\boldsymbol{+(}\boldsymbol{R}_{\boldsymbol{ct}}\boldsymbol{\parallel}\text{CPE}\boldsymbol{)}$**.**

| **Bias (mV)** | $\mathbf{R}_{\mathbf{s}}$**(Ω)** | $\mathbf{R}_{\mathbf{ct}}$ **(kΩ)** | **Q (F·s⁽ⁿ-¹⁾)** | **n** |
| --- | --- | --- | --- | --- |
| 0 | 22.19 | 1435 | 0.1763 | 0.9515 |
| 100 | 21.82 | 1433 | 0.1686 | 0.9489 |
| 200 | 27.43 | 1575 | 0.1495 | 0.9186 |
| 300 | 26.94 | 3572 | 0.1538 | 0.9463 |
| 400 | 28.16 | 4885 | 0.1525 | 0.9541 |
| 500 | 38.94 | 7660 | 0.1313 | 0.9192 |

Table S4 - Functional annotation of putative voltage-gated ion channels in *P. tricornutum*

| **Gene ID** | **PFAM domain** | **Biological process** |
| --- | --- | --- |
| Phatr3_J38095 | Voltage gated chloride channel | Transmembrane transport |
| Phatr3_J39421 | Voltage gated chloride channel | Transmembrane transport |
| Phatr3_J42513 | Voltage dependent anion channel | NA |
| Phatr3_J43785 | Voltage gated chloride channel | Transmembrane transport |
| Phatr3_J46097 | CBS domain; Voltage gated chloride channel | Transport |
| Phatr3_J47784 | Voltage gated chloride channel | Transmembrane transport |
| Phatr3_J52412 | CBS domain; Voltage gated chloride channel | Transport |
| Phatr3_Jdraft1654 | Ion transport protein; Ion transport protein;Kv2 voltage-gated K+ channel | Calcium ion transmembrane transport |
| Phatr3_EG00842 | Voltage gated chloride channel | Transmembrane transport |
| Phatr3_EG01952 | Voltage gated chloride channel | Transmembrane transport |
| Phatr3_J28245 | CBS domain; CBS domain; Voltage gated chloride channel | Transport |
| Phatr3_J54164 | Ion transport protein | Transport |
| Phatr3_J43828 | Ion transport protein | Transport |
| Phatr3_J43878 | Ion transport protein | Transport |
